# Crosstalk between hypoxia-sensing ULK1/2 and YAP-driven glycolysis fuels pancreatic ductal adenocarcinoma development

**DOI:** 10.7150/ijbs.60018

**Published:** 2021-07-05

**Authors:** Yu Jia, Hui-Yan Li, Ying Wang, Jue Wang, Jing-Wen Zhu, Yan-Yan Wei, Lu Lou, Xing Chen, Shi-Jing Mo

**Affiliations:** 1 Cancer Research Center, Tongji Hospital, Tongji Medical College, Huazhong University of Science and Technology, Wuhan 430030, Hubei, P.R.China.; 2 General Surgical Laboratory, The First Affiliated Hospital, Sun Yat-Sen University, Guangzhou, 510080, Guangdong, P.R.China.; 3 Department of Surgery, Huashan Hospital, Fudan University, Shanghai 200040, P.R.China.; 4 Department of Pathology, The First Affiliated Hospital, Sun Yet-Sen University, Guangzhou 510080, Guangdong, P.R.China.

**Keywords:** Hypoxia sensing, Unc-51 like kinase 1 and 2, Yes-associated protein, Phosphorylation, Glycolysis

## Abstract

Autophagy and glycolysis are two catabolic processes that manipulate pancreatic ductal adenocarcinoma (PDAC) development in response to hypoxia sensing, yet the underlying mechanism of how they are interlinked remain elusive.

**Methods:** The functional roles of Unc-51 like kinase 1 and 2 (ULK1/2) in pyruvate kinase M2 (PKM2) transcription and glycolysis under hypoxia were assessed by chromatin immunoprecipitation, luciferase reporter, glucose consumption and lactate production assay. Co-immunoprecipitation, cellular ubiquitination, His-pulldown, *in vitro* protein kinase assay, immunofluorescence, immunohistochemistry, CRISPR technology, in silico studies were adopted to determine the molecular mechanism. Correlation analyses were performed in KPC (*Pdx1*-Cre; LSL-Kras^G12D/+^; Trp53^fl/+^) mice and clinical samples from PDAC patients. Therapeutic potential of ULK1/2 inhibitor and 2-deoxyglucose (2-DG) or 3-bromopyruvate (3-BP) was evaluated in cell-derived xenograft (CDX) and the patient-derived xenograft (PDX) models of nude mice.

**Results:** ULK1/2, but not ULK3, augments hypoxic glycolysis in PDAC cells mediated by PKM2 independent of BCL2/adenovirus E1B 19 kDa interacting protein 3 (BNIP3). Mechanistically, hypoxia stimulates ULK1 to translocate into nucleus, where it interacts with and phosphorylates yes-associated protein (YAP) at Ser227, resulting in YAP stabilization through blockade of ubiquitin-proteasome system (UPS), which in turn facilitates PKM2 transcription, glycolysis, cell proliferation *in vitro* as well as PDAC growth in mice. ULK1/2 is positively correlated with YAP and PKM2 in tumor tissues from KPC mice and clinical samples from PDAC patients. Pharmacological deactivation of ULK1/2 potentiates the antineoplastic efficacy of 2-DG and 3-BP in CDX and PDX models.

**Conclusion:** Our findings underscore the Ser227 autophosphorylation-dependent nuclear YAP stabilization as a central node that couples ULK1/2-initiated autophagy to hypoxic glycolysis during PDAC development and propose that targeting ULK1/2 combined with 2-DG or 3-BP might be a feasible therapeutic strategy against PDAC.

## Introduction

Pancreatic ductal adenocarcinoma (PDAC) is one of the most aggressive human malignancies worldwide, characterized by geographic variation, poor prognosis and high mortality 1. Despite considerable progress concerning therapeutic approaches has been achieved in the past decades, PDAC remains a lethal disease with 5 year disease-free survival rates of less than 6% after initial diagnosis 2. Intratumoral hypoxia sensing, in which hypoxia-inducible factor 1α (HIF-1α) serves as the primary transcription factor (TF) engaging in multiple oncogenic processes, plays a principal role in adapting cancer cells to a state favorable for surviving and maintaining distinct malignant traits as a result of metabolic reprogramming or genetic alterations^3^. Herein, a better understanding of the regulatory mechanisms underlying hypoxia sensing would lay framework for developing innovative and more effective therapies for clinical PDAC.

Aberrant activation of yes-associated protein (YAP), the transcriptional coactivator in Hippo pathway, has been implicated in a large number of human cancers including lung, breast, skin, liver cancer and PDAC 4, 5. The evolutionarily conserved YAP protein shuttles between cytoplasm and nucleus in a context-dependent manner 6. Nuclear localization enables YAP to coordinate transcription of downstream oncogenes and drive tumorigenesis. Indeed, autophosphorylation at Y357 leads to assembly of YAP with T-box transcription factor 5 (TBX5) in nucleus, which in turn promotes the β-catenin-mediated tumor formation 7. Tumor cells harboring 5'-adenosine monophosphate-activated protein kinase (AMPK) phosphorylation-deficient YAP mutant display increased nuclear YAP localization and transcription of *SLC2A3* gene across the plasma membranes 8. The hypoxia-activated siah E3 ubiquitin ligase 2 (SIAH2) triggers nuclear YAP translocation through degrading large tumor suppressor kinase 1/2 (LATS1/2) and accelerate tumorigenicity 9. Our recent study demonstrated that nuclear YAP cooperates with HIF-1α to transactivate pyruvate kinase M2 (PKM2), thereby contributing to hypoxia-dependent glycolysis and tumor growth in PDAC cells 10. However, the precise mechanism of how hypoxia sensing regulates nuclear YAP is hitherto longstanding question with no clear answer.

Similar to aberrant YAP activation, autophagy in certain types of cancer is thought to be a supporting manner to ensure fuel availability and tumor development 11-13. High expression of key component markers of autophagy-lysosome pathway has been observed in PDAC and correlated with worse survival in clinical patients 14. Targeting autophagy-lysosome pathway sensitizes PDAC cells to chemotherapy, ionizing radiation and replication stress 15, 16. Autophagy is initiated by a double-membraned autophagosome consisting of Unc-51 like kinase 1 (ULK1), ULK2 and their associated subunits, Atg13 and RB1CC1/FIP200 17, 18. ULK1/2 increases autophagy flux via inducing substrate phosphorylation 19. Nevertheless, biochemical events that connect ULK1/2 with PDAC development have not yet been identified and our understanding of whether the ULK1/2-initiated autophagy could intersect with glycolysis in the setting of oxygen deprivation remains elusive.

In the present study, we demonstrate that depletion of ULK1/2, but not ULK3, impair PKM2-mediated glycolysis in PDAC cells independent of BNIP3 under hypoxic conditions. Mechanistically, hypoxia stimulates ULK1 to translocate into nucleus, where it interacts with YAP and phosphorylates YAP at Ser227. This event subsequently dismantles the Lys (K)48-linked polyubiquitination of YAP, leading to YAP stabilization and facilitating YAP recruitment at PKM2 gene promoter together with HIF-1α, for which PKM2 transcription is required. ULK1/2-YAP axis is essential for the hypoxic glycolysis and cell proliferation *in vitro* and PDAC growth in mice. Positive correlation of ULK1/2 with YAP and PKM2 expression is observed in in tumor tissues from KPC (*Pdx1*-Cre; LSL-Kras^G12D/+^; Trp53^fl/+^) mice and clinical samples from PDAC patients. Pharmacological deactivation of ULK1/2 using a small-molecule kinase inhibitor SBI-0206965 synergizes with hexokinase I inhibitor 2-deoxyglucose (2-DG) and hexokinase II inhibitor 3-bromopyruvate (3-BP) to suppress tumor development in both PDAC cell- and patient-derived xenograft models. Our study provide a rationale for an unappreciated role of nuclear YAP stabilization triggered by the ULK1/2-dependent Ser227 phosphorylation in linking autophagy to glycolysis during hypoxia sensing and support further investigation of small-molecule ULK1/2 kinase inhibitor in combination with 2-DG and 3-BP in clinical trials of PDAC patients.

## Materials and Methods

### Reagents, antibodies and clinical specimens

SBI-0206965 (cat. No. HY-16966) was purchased from MedChem Express (MCE, Shanghai, China). 2-DG (cat#D8375) and 3-BP (cat#D16490) were obtained from Sigma-Aldrich, Inc. (St. Louis, MO, USA). Glucose Uptake Colorimetric Assay Kit (cat#K676) and Lactate Colorimetric Assay Kit (cat#K627) were purchased from BioVision (Milpitas, CA, USA). Lipofectamine 2000 and antifade reagent with 4', 6-diamidino-2-phenylindole (DAPI) were from Invitrogen (Carlsbad, CA). Annexin V-FITC/PI Apoptosis Detection Kit was from KeyGene Biotech (Nanjing, China). Primary antibodies for western-blotting, co-immunoprecipitation (co-IP), *in vitro* kinase and cellular ubiquitination assays are as the followings: anti-ULK1 (1:1000 dilution, Cell Signaling Technology, cat#8054), anti-ULK2 (1:1000 dilution, Abcam, cat#ab97695), anti-ULK3 (1:1000 dilution, Abcam, cat#ab219264), anti-HIF-1α (1:1000 dilution, Cell Signaling Technology, cat#36169), anti-BNIP3 (1:1000 dilution, Cell Signaling Technology, cat#44060), anti-PKM2 (1:1000 dilution, Abcam, cat#ab137852), anti-PKM1 (1:1000 dilution, Cell Signaling Technology, cat#7067), anti-YAP (1:1000 dilution, Cell Signaling Technology, cat#14074), anti-phospho-serine (1:500 dilution, Santa Cruz, cat# sc-81514), anti-K48-Ub (1:1000 dilution, Cell Signaling Technology, cat#12805S), anti-K63-Ub (1:1000 dilution, Cell Signaling Technology, cat#12930S), anti-Flag (1:1000 dilution, Biosynthesis, cat#bs-0965R), anti-Lamin B (1:1500 dilution, Cell Signaling Technology, cat#13435), anti-GAPDH (1:1500 dilution, Beyotime, cat#AF1186) and anti-β-actin (1:1500 dilution, Beyotime, cat#AF0003).

The use of human PDAC specimens was evaluated and approved by the Institutional Research Ethics Committee of Sun Yat-Sen University (SYSU), and written informed consent was obtained from all participants or their appropriate surrogates. We obtained 95 paraffin-embedded tumor specimens from clinical PDAC patients, which were histopathologically and clinically diagnosed at the First Affiliated Hospital of SYSU during 2013 and 2015. We also collected six cancerous tissues of patients with PDAC, which were immediately confirmed by pathological evaluation after surgical resection and stored frozen in liquid nitrogen for further analyzing ULK2, YAP and PKM2 protein expression.

### Mice

The *Pdx1*-Cre mice (B6.FVB-Tg(Ipfl-cre)1Tuv, D000123) were acquired from the Nanjing Biomedical Research Institute of Nanjing University (Nanjing, China). The LSL-Kras^G12D/+^;Trp53^fl/+^ mice (B6.129-*Kras^tm4Tyj^ Trp53^tm1Brn^*/J, 032435) were purchased from Jackson Laboratory. The KPC (*Pdx1*-Cre; LSL-Kras^G12D/+^; Trp53^fl/+^) mice which could develop adenocarcinoma in pancreas spontaneously were created by crossing the *Pdx1*-Cre mice with the LSL-Kras^G12D/+^;Trp53^fl/+^ mice. All animals were bred in a specific-pathogen-free animal facility, with the temperature controlled at 24°C and a 12h/12h light/dark cycle at the Animal Experiment Center of Huazhong University of Science and Technology (HUST). All animal studies were conducted with the approval of the Institutional Animal Care and Use Committee (IACUC) guidance of HUST.

### Cell culture

Human SW-1990 PDAC cells were cultured according to previous reports 10, 20. Human HepG-2 hepatocellular carcinoma (HCC), MCF-7 breast cancer (BC) and PL45 cells from American Type Culture Collection (Manassas, VA, USA) were grown in high-glucose DMEM (Gibco, Carlsbad, USA) containing 10% FBS, 100 U/mL penicillin and 100 mg/mL streptomycin at 37°C in 5% CO_2_ at a humidified atmosphere. Pancreatic patient-derived cancer cells (PDC#0183) were isolated from primary patient-derived pancreatic ductal adenocarcinoma xenograft and cultured in complete RPMI1640 medium plus 10 ng/mL EGF and 1% insulin-transferrin-selenium (ITS). Physical hypoxia was performed in the indicated cancer cells as previously described 10.

### Plasmids

The pGL3-luciferase reporter gene plasmid containing the full-length promoter region of PKM2 (Promega, Madison, WI) was constructed as described in previous publication 10. The plasmid encoding human wild-type YAP was amplified by PCR and subcloned into the pFlag-CMV^TM^-2 vector (Sigma, St Louis, MO). His-tagged ULK1 was developed using pET-22b (+) vector (EMD Biosciences, Novagen). YAP S227A and S227E mutants were generated using the QuickChange^®^ Site-Directed Mutagenesis Kit (Agilent Technologies, Santa Clara, CA) according to the manufacturer's instructions. The pLV-puromycin lentivirus expressing ULK1 or ULK2 was generated by co-transfection with VSVg and Δ8.9 packaging plasmids in 293T cells. Commercial short hairpin RNA (shRNA) targeting ULK1 (sc-44182-SH), ULK2 (sc-44183-SH) were ordered from Santa Cruz Biotechnology (Santa Cruz, CA). PKM2 shRNA (cat#42516) was from Addgene (Cambridge, MA). The primers for gene cloning were as below:

YAP: 5′-GAATTCAGGTTGGGAGATGGCAAAGA-3′ (forward). 5′-CCGCGGTATTCTGCTGCACTGGTGGA-3′ (reverse). YAP-S227A: 5′-GAATTCCAGAATATGATGAACGCGGCTTCAGGTCCTC-3′ (forward). 5′-CCGCGGGAGGACCTGAAGCCGCGTTCATCATATTCTG-3′ (reverse). YAP-S227E: 5′-GAATTCGCAGAATATGATGAACGAGGCTTCAGGTCCTCTTC-3′ (forward). 5′-CCGCGGGAAGAGGACCTGAAGCCTCGTTCATCATATTCTGC-3′ (reverse).

### Transfection

The protocols for siRNA, shRNA and plasmid transfection were described in previous studies 10, 20, 21. For siRNA transfection, cells were seeded in six-well plates for 12h before transfection. Afterwards, Opti-MEM medium mixing with siRNA and Lipofectamine 2000 reagent (Invitrogen, Carlsbad, CA) was added to each well. Medium was then replaced with fresh medium 6h later and cells were harvested for further analyses after 48 hours. For shRNAs or plasmids transfection, cells were seeded to 80% confluent at the time of transfection. The indicated shRNAs or plasmids were mixed with Lipofectamine 2000 and diluted in DMEM. The mixtures were then incubated for 5 min at room temperature and added to cell cultures of each dish, followed by harvesting the transfected cells 48h later. For lentivirus transfection, PDAC cells were incubated in lentivirus-containing medium with 10 μg/mL polybrene (EMD Millipore, Billerica, MA, USA), stable transfectants were selected by puromycin (1μg/mL) for two weeks after transfection.

### Generation of YAP KO cells by CRISPR-Cas9 gene editing

Commercial human YAP1 sgRNAs (HCP255077-SG01-3) purchased from GeneCopoeia (Rockville, USA) were used to generate YAP knockout (KO) cells. In brief, PDAC cells were cultured in 6-well plates and transfected with YAP1 sgRNAs. Cells were trypsinized 48h later and 200 μg/mL hygromycin was added into the medium for selection of the hygromycin-resistant cells. Clones derived from YAP KO cells were obtained by serial dilutions in a 96-well plate and further confirmed by western blotting.

### His-pulldown and* in vitro* kinase assays

His-pulldown assay was performed as previously described 10. Nuclear lysates from the hypoxia-stimulated PDAC cells expressing Flag-tagged wild-type YAP were incubated with 100 ng of His fusion proteins immobilized on Ni^2+^-nitrilotriacetic acid (Ni-NTA)-sepharose beads overnight at 4 °C. The beads were then washed with lysis buffer for three times, and the bound proteins were subjected to sodium dodecyl sulfate-polyacrylimide gel electrophoresis (SDS-PAGE) and blotted with the indicated antibodies.

The *in vitro* kinase reactions were carried out as described before 10. In brief, Flag-tagged wild-type YAP or mutant YAP S227A was transfected into PDAC cells, immunoprecipitated by an anti-Flag antibody and eluted from protein A/G-agarose beads (Cwbiotech, Beijing, China) using Flag peptides. The immunoprecipitated Flag-tagged wild-type YAP or mutant YAP S227A were then incubated with purified His-ULK1 fusion proteins in the presence of 200 µM ATP in a kinase buffer for 30 min. Reaction products were subjected to SDS-PAGE and blotted with the anti-phospho-Ser antibody.

### Cellular ubiquitination and cycloheximide (CHX) pulse-chase assays

Cellular ubiquitination assay was done essentially according to previous study with some modifications 20. Hypoxia-stimulated PDAC cells were transfected with the indicated shRNAs or plasmids in the presence or absence of His-tagged Ub for 48h and lysed by the denatured buffer containing 6 mol/L guanidine-HCl, 0.1 mol/L Na_2_HPO_4_/NaH_2_PO_4_, 10 mmol/L imidazole. The cell lysates were then incubated with Ni-NTA-sepharose beads for 6h to pull down the ubiquitinated proteins, followed by washing and subjecting to western-blotting analyses with the indicated antibodies. To examine the K48- and K63-linked YAP polyubiquitylation, cell lysates from the hypoxia-stimulated SW-1990 cells with ULK1/2 shRNA transfection were lysed by radio-immunoprecipitation assay (RIPA) buffer (Cwbiotech, Beijing, China) containing protease inhibitors and phosphatase inhibitor cocktail. Afterwards, lysates were incubated with anti-YAP antibody and protein A/G-agarose beads for immunoprecipitating YAP protein. The immunoprecipitates (IPs) were then subjected to western-blotting with either an anti-K48 or an anti-K63 ubiquitin antibody to visualize the polyubiquitylated YAP protein bands.

Cycloheximide (CHX) pulse-chase experiments were performed to detect the turnover of YAP protein. Generally, PDAC cells were seeded on 12-well plate at a density of 1 × 10^5^ cells per well. After culturing overnight, cells were transfected with the indicated shRNAs or plasmids as desired. Forty-eight hours after transfection, cells were treated with 20 μg/mL CHX dissolved in dimethyl sulfoxide (DMSO), and total protein lysates were collected at different time points and subjected to western-blotting analyses.

### Co-immunoprecipitation and western blotting

Co-immunoprecipitation (co-IP) and western blotting (WB) analyses were conducted according to the detailed procedures in previous studies 10, 20, 21. Briefly, proteins were extracted from cultured cells using RIPA buffer containing 50 mM Tris [PH7.4], 150 mM NaCl, 1% NP-40, 0.25% sodium deoxycholate and protease inhibitors. Cell extractions were centrifuged with 15,000 g speed for 15 min at 4°C and the supernatants were subjected to immunoprecipitation using the indicated primary antibodies and protein A/G-agarose beads before electrophoresis on SDS-polyacrylamide gels. The bands of co-IP and WB were visualized by western chemiluminescent HRP substrate kit (PPLYGEN, Beijing, China).

### Immunofluorescence analysis

Immunofluorescence (IF) was carried out using standard methods as described previously 10, 20. In brief, the indicated PDAC cells were fixed in 4% paraformaldehyde at room temperature for 15 min and permeabilized in 0.5% Triton X-100 in PBS for 10 min. After being blocked with PBS solution including 3% donkey serum, 1% BSA and incubated with primary antibodies for 1h, cells were incubated with Alexa Fluor^®^ 594 and 488 conjugate secondary antibodies (1:500, Cell Signaling Technology) at 37°C for 1h. Nuclei were stained with DAPI and pictures were viewed with an IX71 microscope (Olympus, Japan).

### Chromatin immunoprecipitation (ChIP) assay

The ChIP assay was performed using EZ-Magna ChIP kit (Millipore, Billerica, MA) according to the manufacturer's protocol. Briefly, hypoxia-stimulated PDAC cells transfected with the indicated shRNAs or plasmids were crosslinked in 1% formaldehyde solution for 10 min at room temperature, followed by sonicating to generate DNA fragments with the average size below 1,000 base pairs bp by lengths. Two micrograms of anti-IgG, anti-YAP or anti-HIF-1α was used for each immunoprecipitation, respectively. The immunoprecipitates were eluted and reverse crosslinked, after which the DNA fragments were purified. Immunoprecipitated and input DNAs were subjected to semiquantitative PCR analysis. The ChIP primers used for amplification of PKM2 gene promoter were described in previous study 10.

### Luciferase reporter assay

Dual-luciferase reporter assays were carried out using Dual Luciferase Reporter Assay Kit (Promega, Madison, WI) as described in previous study^10^. In brief, the promoter fragment of PKM2 gene was amplified by PCR and subcloned into pGL3 luciferase reporter plasmid. PDAC cells cultured in 24-well plates were transfected with 100 ng of pGL3 luciferase reporter plasmid and 1 ng of pRL-TK Renilla plasmid using Lipofectamine 2000 (Invitrogen). Luciferase activity was determined according to the manufacturer's instructions and three independent experiments were performed. Transfection efficiency was normalized on the basis of the Renilla luciferase activity.

### RNA extraction and RT-qPCR

Real-time quantitative PCR (RT-qPCR) experiments were carried out as previously described 10, 20, 21. In brief, RNA extracted from the indicated PDAC cells by Trizol (Invitrogen) was reverse transcribed to cDNA with PrimeScript^®^RT reagent Kit (Takana, Dalian, China) using Super Array PCR master mix (SuperArray Bioscience, Frederick, Maryland, USA). Quantitative real-time PCR was performed with the double-stranded DNA dye SYBR Green PCR Mastermix in Takana SYBR^®^ Primix Ex Taq^TM^Kit (Takana, Dalian, China) on an Applied Biosystems 7900HT Fast Real Time Machine (Applied Biosystems). The following primer pairs were used: PKM1 sense, 5′-CTGGAGAAACAGCCAAAGG-3′ and PKM1 antisense, 5′-GCCAGACTCCGTCAGAACTA-3′; PKM2 sense, 5′-ATGTCGAAGCCCCATAGTGAA-3′ and PKM2 antisense, 5′-TGGGTGGTGAATCAATGTCCA-3′; YAP1 sense, 5′-TAGCCCTGCGTAGCCAGTTA-3′ and YAP1 antisense, 5′-TCATGCTTAGTCCACTGTCTGT-3′; ULK1 sense, 5′-GGCAAGTTCGAGTTCTCCCG-3′ and ULK1 antisense, 5′-CGACCTCCAAATCGTGCTTCT-3′; ULK2 sense, 5′-TGGAGACCTCGCAGATTATTTGC-3′ and ULK2 antisense, 5′-CTGTGCAGGATTCGCATGG-3′; GAPDH sense, 5′-GGAGCGAGATCCCTCCAAAAT-3′ and GAPDH antisense, 5′- GGCTGTTGTCATACTTCTCATGG-3′.

### Glucose consumption and lactate production assay

Glucose uptake and lactate production of the indicated cells were measured using Glucose Uptake Colorimetric Assay Kit and Lactate Colorimetric Assay Kit from BioVision (Milpitas, CA, USA) according to the manufacturers' recommendations as described in previous publication 10.

### Clonogenic survival assay

Clonogenic survival assays were performed as previously described 10, 20, 21. The indicated PDAC cells growing at 80% confluence were trypsinized and transferred into fresh medium in a single cell suspension. One thousand cells were plated per well in six-well plates and incubated for ten days with complete culture medium containing 10% FBS before fixing and staining with 0.5% crystal violet solution (0.5% w/v crystal violet powder, 80% v/v H_2_O and 20% v/v methanol). The number of colonies was counted by ImageJ software according to the user manual.

### Viability assay

Relative viability of PDAC cells in response to 2-DG or 3-BP and SBI-0206965 treatment was assessed by 3-(4,5-dimethylthiazol-2-yl)-2,5-diphenyltetrazolium bromide reduction (MTT) assay which determines the number of viable cells based on the relative amount of formazan in cultures. Briefly, cells were seeded in 96-well plates at a density of 1×10^4^ per well. Increasing doses of drugs in various combinations—2-DG or 3-BP alone or in combination with SBI-0206965—were added into the plates after 24 hours. In all cases, DMSO controls were run in parallel to the drug treatments. At the end of the experiment, 20 μL of an MTT (5 mg/mL) was added and the plates were incubated at 37°C for 4h. To dissolve formazan, 150 mL dimethyl sulfoxide was added and then the absorbance was detected at 490 nm by spectrometer (Wellscan MK3; Labsystems Dragon).

### Flow cytometry with Annexin V/PI staining

For Annexin-V/Propidium iodide (PI) double staining, PDAC cells were seeded in 24-well plates and underwent 2-DG or 3-BP alone or in combination with SBI-0206965 treatments as indicated, and then harvested, washed and incubated with binding buffer containing 5 μL Annexin V-FITC and 5 μL PI at 37°C. Twenty minutes later, cells suspensions were subjected to fluorescence activated cell sorter (FACS) analysis.

### Subcutaneous xenograft experiments

Both the PDAC cell-derived xenograft (CDX) and the patient-derived xenograft (PDX) experiments were performed in accordance with established guidelines as reported previously 10, 20. To establish CDX models, the indicated SW-1990 cells (1×10^7^) were subcutaneously injected into the right flank of BALB/c nude mice (athymic nu/nu, 6-week old, male) housed in a pathogen-free facility. For establishment of PDX models, tumor specimens from a PDAC patient were cut into small pieces (less than 1mm^3^) and mixed with Matrigel (100 μL per sample) (BD Biosciences) at room temperature for 20 seconds prior to subcutaneous inoculation. In both xenograft models, tumor growth was measured by a caliper and volume was calculated with the equation *V* (mm^3^) = *a*× *b*^2^/2, where *a* is the largest diameter and *b* is the perpendicular diameter. To evaluate therapeutic effectiveness, 2-DG or 3-BP at a dose of 0.5 g/kg or 1 mg/kg per animal was intraperitoneally (i.p.) injected into nude mice for 5 day-intervals from seven days after xenotransplantation, and SBI-0206965 was administered at a dose of 50 mg/kg per animal. In the combined therapy of 2-DG or 3-BP with SBI-0206965, the same doses of each drug per animal were administered by i.p. injection, respectively. The mice were killed at the indicated times after inoculation and the xenografts were then excised, weighed and subjected to histopathological examination.

### Mice blood analysis

Whole blood (200 μL) was collected from the orbital sinus of mice using a microhematocrit blood tube at the end point of drug experiments. Blood samples were stored in the heparin-coated tubes for hematology study of platelets (PLT) count and biochemical detection of alanine transaminase (ALT) and serum creatinine (Scr) levels to evaluate therapeutic tolerability at Department of Laboratory Medicine in Tongji Hospital of HUST.

### Immunohistochemical staining

The immunohistochemical stainings were performed on 5μm sections as previously described 10, 20, 21. In brief, the sections were placed on poly-lysinecoated slides, deparaffinized in xylene, rehydrated through graded ethanol, quenched for endogenous peroxidase activity in 2% hydrogen peroxide and processed for antigen retrieval by microwave heating for 10 min in citrate buffer. Sections were then incubated with the primary antibodies diluted in PBS (1: 250) at 4°C overnight. After that, the Dako ChemMate^TM^ EnVision^TM^ Detection Kit peroxidase/diaminobenzidine.

(DAB) was utilized to perform immunohistochemical staining and sections were counterstained with hematoxylin. The images were captured using Nikon Ti-S microscope equipped with a digital camera system (Nikon, Japan). The staining was evaluated and scored independently by two experienced observers who were blinded to the study. The IHC score was calculated through multiplying the percentage of stained cells (0%-100%) by the intensity of the staining (1, low and 2, strong). Score<0.5 and score≥0.5 was separately defined as low and high expression of ULK2, YAP and PKM2 in the correlation analysis.

### In silico studies

Microarray-based data that compared ULK1/2, YAP1 and PKM2 expression between pancreatic cancer and normal pancreatic tissues were identified by searching the Oncomine cancer profiling database. The Badea, Ishikawa and Segara datasets were chosen for the comparative analyses 22-24. Gene expression dataset (GSE15471) including 36 PDAC tumors and matching normal pancreatic tissue samples was downloaded from Gene Expression Omnibus (GEO) and the raw data were subjected to intensity comparison after log transformation. Both YAP1 and PKM signatures were constructed on genes whose expressions are positively correlated with YAP1 or PKM in the Cancer Cell Line Encyclopedia (CCLE) dataset of cbioportal (http://www.cbioportal.org/). Based on Pearson's correlation coefficient ≥ 0.3 as cutoff, 399 genes were selected as YAP1 signature and 458 genes were selected as PKM signature. Genes of the two signatures were imported into gene ontology (GO) analysis using Metascape (version 3.0)^25^ and gene set enrichment analysis (GSEA) utilizing GSEA desktop v3.0 (Broad Institute).

### Gene set enrichment analysis

Microarray data of a subset of PDAC specimens in which ULK2 mRNA expression is available were downloaded from the GEO database using the accession number indicated in Figure 6G and were incorporated into BROAD javaGSEA standalone version (http://www.broadinstitute.org/gsea/downloads.jsp) to determine the correlation of ULK2 with YAP1 and PKM signature, respectively. The metric for ranking genes in GSEA was set as 'Pearson' and the other parameters were set to their default values.

### Statistical analysis

Statistical analyses were performed using the SPSS 17.0 software package. All experiments *in vitro* were performed independently for at least three times and results are expressed as mean ± standard deviation. Statistical comparison between two groups was assessed using unpaired two-sided Student's *t* test with unequal variance assumption. Bonferroni *post-hoc t* tests were performed to make statistical comparisons in multigroup analysis after a significant result was obtained using ANOVA. The Pearson's or Spearman's rank correlation coefficient was used to clarify the correlation of ULK2 expression with YAP and PKM2 levels. A *P* value < 0.05 was considered statistically significant.

## Results

### Kinase activity of ULK1/2 is instrumental for hypoxic glycolysis mediated by PKM2 independent of BNIP3

**Figure 1:**
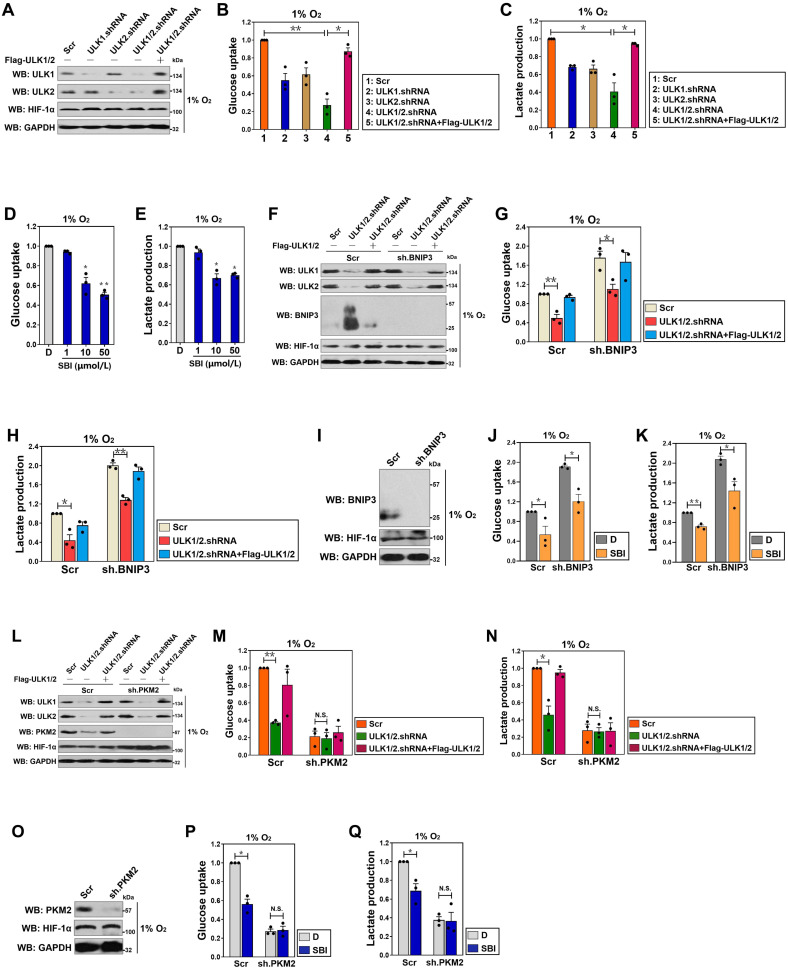
** Kinase activity of ULK1/2 is instrumental for hypoxic glycolysis mediated by PKM2 independent of BNIP3.** (A) Western-blotting comparing the levels of ULK1 and ULK2 expression in SW-1990 cells transfected with ULK1, ULK2 and ULK1/2 shRNA in the presence or absence of Flag-tagged wild-type ULK1/2 expression under hypoxia, respectively. GAPDH was used as internal control of cytoplasmic lysates. Scr, scrambled shRNA; WB, western-blotting. (B and C) Glucose consumption (B) or lactate production (C) of SW-1990 cells transfected with ULK1, ULK2 and ULK1/2 shRNA in the presence or absence of Flag-tagged wild-type ULK1/2 expression under hypoxia. Data are expressed as mean ± s.d. of three independent experiments. *P<0.05 and **P<0.01. Two-sided ANOVA with Bonferroni post hoc *t* test correction was used to calculate the *P* value. (D and E) Glucose consumption (D) or lactate production (E) of SW-1990 cells treated with SBI-0206965 (SBI) at the indicated concentrations under hypoxia. Data are expressed as mean ± s.d. of three independent experiments. *P<0.05 and **P<0.01 versus D. D, DMSO. Two-sided ANOVA with Bonferroni post hoc *t* test correction was used to calculate the *P* value. (F) Western-blotting detecting the amount of ULK1, ULK2 and BNIP3 expression in SW-1990 cells transfected with BNIP3 shRNA (sh.BNIP3) in the presence or absence of ULK1/2 shRNA expression or ULK1/2 shRNA plus Flag-tagged wild-type ULK1/2 coexpression under hypoxia. (G and H) Glucose consumption (G) or lactate production (H) of SW-1990 cells transfected with BNIP3 shRNA (sh.BNIP3) in the presence or absence of ULK1/2 shRNA expression or ULK1/2 shRNA plus Flag-tagged wild-type ULK1/2 coexpression under hypoxia. Data are expressed as mean ± s.d. of three independent experiments. *P<0.05 and **P<0.01. Two-sided ANOVA with Bonferroni post hoc *t* test correction was used to calculate the *P* value. (I) Western-blotting assessing the amount of BNIP3 expression in SW-1990 cells with BNIP3 shRNA (sh.BNIP3) transfection. (J and K) Glucose consumption (J) or lactate production (K) of SW-1990 cells transfected with BNIP3 shRNA (sh.BNIP3) in the presence or absence of 50 μmol/L SBI-0206965 (SBI) treatment under hypoxia. Data are expressed as mean ± s.d. of three independent experiments. *P<0.05 and **P<0.01. Two-sided Student's t test was used to calculate the *P* value. (L) Western-blotting detecting the amount of ULK1, ULK2 and PKM2 expression in SW-1990 cells transfected with PKM2 shRNA (sh.PKM2) in the presence or absence of ULK1/2 shRNA expression or ULK1/2 shRNA plus Flag-tagged wild-type ULK1/2 coexpression under hypoxia. (M and N) Glucose consumption (M) or lactate production (N) of SW-1990 cells transfected with PKM2 shRNA (sh.PKM2) in the presence or absence of ULK1/2 shRNA expression or ULK1/2 shRNA plus Flag-tagged wild-type ULK1/2 coexpression under hypoxia. Data are expressed as mean ± s.d. of three independent experiments. *P<0.05 and **P<0.01. Two-sided ANOVA with Bonferroni post hoc *t* test correction was used to calculate the *P* value. N.S., no significant. (O) Western-blotting examining the amount of PKM2 expression in SW-1990 cells with PKM2 shRNA (sh.PKM2) transfection. (P and Q) Glucose consumption (P) or lactate production (Q) of SW-1990 cells transfected with PKM2 shRNA (sh.PKM2) in the presence or absence of 50 μmol/L SBI-0206965 (SBI) treatment under hypoxia. Data are expressed as mean ± s.d. of three independent experiments. *P<0.05. Two-sided Student's t test was used to calculate the *P* value.

To date, the molecular mechanism linking autophagy and glycolysis during PDAC development in response to hypoxia sensing remains unclear. To investigate the intrinsic relationship between autophagy and glycolysis in context of hypoxia, we transfected small interfering RNA (siRNA) duplexes targeting three isoforms of ULK (ULK1, ULK2 and ULK3) into SW-1990 cells (S-1 for brevity) (Figure S1A), and then stimulated them with hypoxia. Glucose uptake and lactate production, two parameters of glycolysis, decreased by average 2.0- and 1.3-fold in the ULK1 siRNA-transfected cells, and by about 1.5- and 1.4-fold in the ULK2 siRNA-transfected cells upon hypoxia stimuli, respectively, compared with those in the untransfected or the control siRNA-transfected cells (*P* < 0.05, Figure S1B and C). However, no significant alterations of glucose uptake and lactate production were detected in the cells transfected with ULK3 siRNA under the same conditions. Double silencing of ULK1 plus ULK2 (ULK1/2) using RNAi, but not that of ULK1 plus ULK3 (ULK1/3) or ULK2 plus ULK3 (ULK2/3), additively decreased the hypoxia-stimulated glucose uptake and lactate production (*P* < 0.05 and* P* < 0.01). The blockade of hypoxia-stimulated glycolysis by ULK1/2 deficiency is a common feature not restricted to PDAC as such phenomena were also observed in hepatocellular carcinoma (HCC) HepG-2 and breast cancer (BC) MCF-7 cells (*P* < 0.01 and* P* < 0.05, Figure S1D-I), implying that ULK1/2 is indispensable for the hypoxic glycolysis of solid cancer cells. The requirement of ULK1/2 for hypoxic glycolysis was further confirmed by S-1 cells bearing ULK1, ULK2 or ULK1/2 short hairpin RNA (shRNA), which displayed reduced glucose uptake and lactate production in comparison with the parental cells bearing scrambled shRNA (Scr) under hypoxia (*P* < 0.01 and* P* < 0.05, Figure 1A-C). These data strongly indicate that ULK1/2 contributes to hypoxic glycolysis in PDAC cells.

To exclude the possibility of an off-target impact of ULK1/2 on the hypoxic glycolysis, we tested whether the inhibitory role of ULK1/2 deficiency in glucose uptake and lactate production could be rescued by re-expression of ULK1/2. To approach this, we ectopically expressed Flag-tagged wild-type ULK1/2 in the ULK1/2 shRNA-transfected S-1 cells (Figure 1A) and examined the resultant effects. Figure 1B and C revealed that exogenous introduction of Flag-ULK1/2 in the ULK1/2 shRNA-transfected cells readily rescued the glucose uptake and lactate production impaired by ULK1/2 shRNA under hypoxia (*P* < 0.05), ruling out possible off-target impact of ULK1/2 deficiency.

In order to determine kinase activity of ULK1/2 toward the hypoxic glycolysis, an ULK1/2-null PDAC cell line, PL45 (P-45), was transfected with either wild-type (WT) or kinase inactive (KI) ULK1 before being exposed to hypoxia. We found that ectopic expression of ULK1-WT, which had little effect on glucose uptake and lactate production under normoxic circumstances, caused a significant augmentation in both cases after hypoxia exposure (*P* < 0.05), while P-45 cells expressing ULK1-KI exhibited comparable magnitude of glucose uptake and lactate production in contrast to the cells expressing empty vector (EV) irrespective of hypoxic status (Figure S1J-L). Pretreatment with SBI-0206965 (SBI), a small-molecule kinase inhibitor of ULK1/2 26, appreciably reduced glucose uptake and lactate production of the hypoxia-stimulated S-1 cells (*P* < 0.05 and* P* < 0.01, Figure 1D and E). These data reinforce the notion that kinase activity of ULK1/2 might be essential for the hypoxic glycolysis of PDAC cells.

ULK1/2 is a known inhibitor of BCL2/adenovirus E1B 19 kDa interacting protein 3 (BNIP3) 27, the atypical BH3-only protein which functions as a tumor suppressor in human cancers 28-30. We thus hypothesized that the contributing role of ULK1/2 in the hypoxic glycolysis is BNIP3 dependent. For this purpose, levels of glucose uptake and lactate production were measured in the hypoxia-stimulated S-1 cells with ULK1/2 shRNA transfection or ULK1/2 shRNA plus Flag-ULK1/2 cotransfection upon concomitant depletion of BNIP3 using shRNA (Figure 1F). Unexpectedly, the ULK1/2 shRNA-inducible suppression of glucose uptake and lactate production upon hypoxia stimuli was unaffected by depletion of BNIP3 (*P* < 0.05 and *P* < 0.01, Figure 1G and H). Meanwhile, there was no obvious alterations in the SBI-reduced glucose uptake and lactate production after depleting BNIP3 during hypoxia (*P* < 0.05, Figure 1I-K). These data indicate that ULK1/2 contributes to hypoxic glycolysis of PDAC cells independent of BNIP3.

The rate-limiting enzyme responsible for catalyzing final step of glycolysis is pyruvate kinase M2 (PKM2) 31. To examine whether the inhibitory role of ULK1/2 deficiency in hypoxic glycolysis is dependent on PKM2, we depleted PKM2 in the ULK1/2 shRNA-transfected or the ULK1/2 shRNA plus Flag-ULK1/2-cotransfected S-1 cells (Figure 1L). Despite ULK1/2 deficiency caused a notable reduction in glucose uptake and lactate production that could be rescued by ectopic expression of ULK1/2 under hypoxia (*P* < 0.01 and* P* < 0.05), it lost the ability to reduce both glucose uptake and lactate production regardless of ULK1/2 re-expression when PKM2 was depleted (Figure 1M and N), indicating that ULK1/2 deficiency suppresses hypoxic glycolysis mainly by regulating PKM2. In support of this, SBI pretreatment successfully decreased glucose uptake and lactate production of the hypoxia-stimulated S-1 cells (*P* < 0.05), but it could no longer to do so after simultaneous depletion of PKM2 (Figure 1O-Q). These data together suggest that ULK1/2 is required for the hypoxic glycolysis mediated by PKM2 in PDAC cells.

### YAP transactivates PKM2 in an ULK1/2-dependent fashion under hypoxia

**Figure 2:**
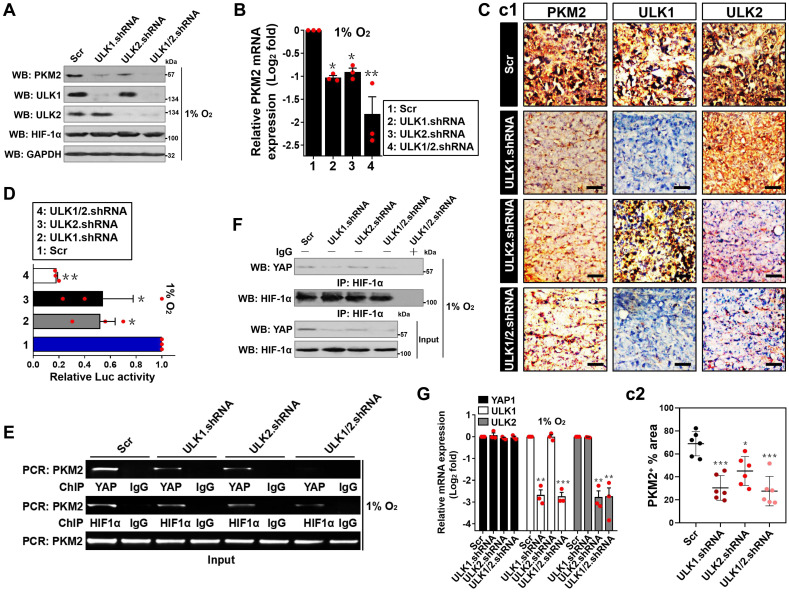
** YAP transactivates PKM2 in an ULK1/2-dependent fashion under hypoxia.** (A) Western-blotting determining the amount of PKM2 expression in SW-1990 cells transfected with ULK1, ULK2 and ULK1/2 shRNA under hypoxia, respectively. Scr, scrambled shRNA. (B) RT-qPCR analyses of PKM2 gene expression in SW-1990 cells transfected with ULK1, ULK2 and ULK1/2 shRNA under hypoxia, respectively. Experiments were performed three times, each with quantitative RT-PCR in technical duplicate and real-time values were normalized to glyceraldehyde 3-phosphate dehydrogenase (GAPDH). Data are expressed as mean ± s.d. *P<0.05 and **P<0.01 versus Scr. Two-sided ANOVA with Bonferroni post hoc *t* test correction was used to calculate the *P* value. (C) Representative IHC staining images (c1) and quantification (c2) for PKM2 staining in the xenografts from mice injected with SW-1990 cells expressing ULK1, ULK2 or ULK1/2 shRNA (*n*=6). Scale bar = 50 μm. (D) Luciferase-reporter PKM2 promoter activity analysis of SW-1990 cells transfected with ULK1, ULK2 and ULK1/2 shRNA under hypoxia, respectively. Data are expressed as mean ± s.d. of three independent experiments. *P<0.05 and **P<0.01 versus Scr. Two-sided ANOVA with Bonferroni post hoc *t* test correction was used to calculate the *P* value. (E) ChIP analysis for YAP and HIF-1α binding to PKM2 gene promoter in SW-1990 cells transfected with ULK1, ULK2 and ULK1/2 shRNA in the presence of hypoxia, respectively. (F) Coimmunoprecipitation assay testing the interaction between nuclear YAP and HIF-1α in SW-1990 cells transfected with ULK1, ULK2 and ULK1/2 shRNA under hypoxia, respectively. (G) RT-qPCR analyses of YAP1 gene expression in SW-1990 cells transfected with ULK1, ULK2 and ULK1/2 shRNA under hypoxia, respectively. Experiments were performed three times, each with quantitative RT-PCR in technical duplicate and real-time values were normalized to glyceraldehyde 3-phosphate dehydrogenase (GAPDH). Data are expressed as mean ± s.d. **P<0.01 and ***P<0.001 versus Scr. Two-sided ANOVA with Bonferroni post hoc *t* test correction was used to calculate the *P* value. (H) Western-blotting comparing the abundance of PKM2 expression in SW-1990 cells with or without ULK1/2 shRNA transfection in the presence or absence of YAP knockout (KO) under hypoxia. (I) RT-qPCR analyses of PKM2 gene expression in SW-1990 cells with or without ULK1/2 shRNA transfection in the presence or absence of YAP knockout (KO) under hypoxia, respectively. Experiments were performed three times, each with quantitative RT-PCR in technical duplicate and real-time values were normalized to glyceraldehyde 3-phosphate dehydrogenase (GAPDH). Data are expressed as mean ± s.d. **P<0.01 versus -. Two-sided Student's t test was used to calculate the *P* value. (J) Luciferase-reporter PKM2 promoter activity analysis of SW-1990 cells with or without ULK1/2 shRNA transfection in the presence or absence of YAP knockout (KO) under hypoxia, respectively. Data are expressed as mean ± s.d. of three independent experiments. **P<0.01 versus -. Two-sided Student's t test was used to calculate the *P* value.

If ULK1/2 has a prinicipal role in the hypoxic glycolysis mediated by PKM2, silencing ULK1/2 would repress PKM2. Indeed, the siRNA-mediated silencing of ULK1, ULK2 or both, rather than ULK3, significantly downregulated the steady-state levels of PKM2 protein under hypoxia, which kinetically correlated with a lower mRNA expression of PKM2 as measured by the real-time quantitative reverse transcriptase-polymerase chain reaction (RT-qPCR) analyses using primers specific for PKM2 mRNA (*P* < 0.05 and* P* < 0.01, Figure S2A and B). In sharp contrast, neither protein nor mRNA levels of PKM1 were influenced after silencing of ULK1, ULK2 or both and ULK3, respectively. Likewise, deactivating ULK1/2 by high dose of SBI in the hypoxia-stimulated cells reduced the amount of PKM2 protein to an extent corresponding to the decrease in its mRNA abundance (*P* < 0.05), while identical deactivation of ULK1/2 had no effect on PKM1 protein and mRNA expression (Figure S2C and D). In accordance with the results obtained from RNA interference and SBI treatment, depletion of ULK1, ULK2 or both using shRNA dramatically decreased the levels of PKM2 protein and mRNA expression in the hypoxia-stimulated S-1 cells (*P* < 0.05 and* P* < 0.01, Figure 2A and B). Introduction of ULK1-WT, but not ULK1-KI, in P-45 cells enhanced protein and mRNA abundance of PKM2 without affecting PKM1 protein and mRNA expression under hypoxia (*P* < 0.05, Figure S2E and F). To evaluate the fundamental role of ULK1/2 on PKM2 expression *in vivo*, we examined PKM2 expression using immunohistochemistry (IHC) in subcutaneous tumor xenografts arised from nude mice that were inoculated with S-1 cells bearing ULK1, ULK2 or ULK1/2 shRNA. As expected, these tumor xenografts exhibited robust diminution of PKM2-positive staining in comparison with their counterparts (Figure 2C). These results validate that ULK1/2 upregulates PKM2 expression under hypoxic circumstances *in vitro* and in tumorigenic microenvironment *in vivo*.

In an attempt to decipher whether ULK1/2 augments PKM2 transcription under hypoxia, we utilized luciferase reporter assay to detect PKM2 promoter activity in the hypoxia-stimulated S-1 cells expressed a pGL3-PKM2-Luc vector containing the full-length PKM2 promoter. We found that PKM2 promoter activity was largely reduced when ULK1, ULK2 or both had been depleted and, to a lesser degree after SBI administration (*P* < 0.05 and* P* < 0.01, Figure 2D; *P* < 0.05, Figure S2G). Given our previous study demonstrated that nuclear YAP interacts with HIF-1α to enhance PKM2 transcription in response to hypoxia 32, we reasoned that ULK1/2 might augment PKM2 transcription via influencing YAP-HIF-1α interaction. To test this possibility, we examined whether ULK1/2 affects the recruitment of YAP and HIF-1α at *PKM2* gene promoter by performing chromatin immunoprecipitation (ChIP) assay in the hypoxia-stimulated S-1 cells transfecting with ULK1, ULK2 or ULK1/2 shRNA. As shown in Figure 2E, the anti-immunoglobulin G (IgG) antibody did not pull down promoter region of *PKM2* gene in any cells tested. Of note, cells lacking ULK1, ULK2 or both were characterized with a profound reduction in recruitment of YAP but not HIF-1α at *PKM2* promoter. Meanwhile, western-blotting (WB) of immunoprecipitated HIF-1α in nuclear extraction (NE) from S-1 cells with an anti-YAP antibody showed that depletion of ULK1, ULK2 or both using shRNA disrupted the interaction between nuclear YAP and HIF-1α at endogenous levels, which, in the case of hypoxia stimuli, was accompanied by a significant decline in YAP protein levels but not its mRNA expression (Figure 2F and G), indicating that the observed disruption of YAP-HIF-1α interaction is probably due to post-translational downregulation of YAP by ULK1/2 deficiency under hypoxia. The essentiality of ULK1/2 for YAP-HIF-1α interaction was also demonstrated by a cell-free assay, where NE from S-1 cells expressing Flag-tagged wild-type YAP were incubated with or without purified ULK1. As shown in Figure S2H, the binding of YAP to HIF-1α became more evident after ULK1 was added to the reaction mixtures, underscoring an important role of ULK1 in facilitating YAP-HIF-1α interaction. Administration of SBI also impaired YAP accumulation in NE (Figure S2I), which may account for the reduction in promoter activity of *PKM2* gene under hypoxia.

To surmise whether the role of ULK1/2 on hypoxic PKM2 transcription is mediated by YAP, we knocked out endogenous YAP protein (termed as eYAP hereafter) using CRISPR-Cas9 genome editing in the hypoxia-stimulated S-1 cells with or without ULK1/2 shRNA transfection. As shown in Figure 2H-J and S2J, depletion of ULK1/2 repressed protein expression, mRNA levels (*P* < 0.01) and promoter activity (*P* < 0.01) of PKM2 but such effects did not occur when eYAP was knocked out (KO). Similar phenomena were observed in the eYAP KO cells administered with SBI (*P* < 0.05, Figure S2K-M), suggesting that ULK1/2 augments PKM2 transcription via YAP during hypoxia.

### ULK1/2 stabilizes nuclear YAP in response to hypoxia

**Figure 3:**
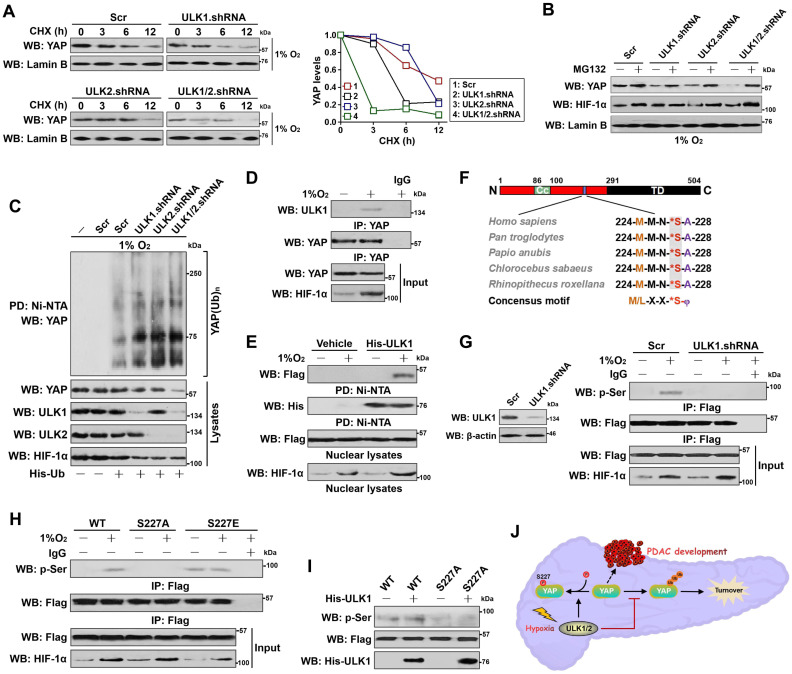
** ULK1 translocates into nucleus and phosphorylates YAP at Ser227 during hypoxia, which leads to YAP stabilization.** (A) CHX pulse-chase experiments comparing the turnover of YAP protein in SW-1990 cells transfected with ULK1, ULK2 and ULK1/2 shRNA in the presence of 20 μg/mL CHX treatment for the indicated times under hypoxia, respectively. (B) Western blotting analyses testing abundance of YAP protein in the hypoxia-stimulated SW-1990 cells transfected with ULK1, ULK2 and ULK1/2 shRNA in the presence or absence of 10 μmol/L MG132 treatment, respectively. (C) Cellular ubiquitination assays examining the poly-Ub levels of YAP in SW-1990 cells transfected with ULK1, ULK2 and ULK1/2 shRNA under hypoxia, respectively. PD, pull-down; Ni-NTA, Ni^2+^-nitrilotriacetic acid (NTA). (D) Coimmunoprecipitation assay evaluating the interaction between ULK1 and nuclear YAP in SW-1990 cells with or without hypoxia stimuli. (E) His-pulldown assay for determination of ULK1-YAP interaction with mixing purified His-tagged ULK1 immobilized on Ni^2+^-nitrilotriacetic acid (NTA)-sepharose beads and nuclear lysates from hypoxia-stimulated SW-1990 cells expressing Flag-tagged wild-type YAP followed by WB analyses of proteins on beads with an anti-Flag antibody. PD, pull-down. (F) Sequence alignment of the evolutionarily conserved ULK1-phosphorylating motif ^224^MMN*SA^228^ peptide in amino acid sequence of YAP protein among human and other primates. (G) Left panel: western-blotting examining the levels of ULK1 protein in the SW-1990 cells transfected with ULK1 shRNA. Right panel: coimmunoprecipitation assay detecting the abundance of Flag-tagged wild-type YAP phosphorylation in hypoxia-stimulated SW-1990 cells in the presence or absence of ULK1 shRNA transfection with an anti-phospho-serine antibody. (H) Coimmunoprecipitation assay comparing the levels of Flag-tagged wild-type YAP (WT), mutant YAP Ser227A (S227A) and Ser227E (S227E) phosphorylation in hypoxia-stimulated SW-1990 cells with an anti-phospho-serine antibody. (I) *In vitro* kinase assay with mixing His-tagged ULK1 protein and IPs of Flag-tagged wild-type YAP (WT) or mutant YAP Ser227A (S227A) followed by WB analyses with an anti-phospho-serine antibody. (J) Schematic defining the role of ULK1/2 in Ser227 phosphorylation and stabilization of nuclear YAP during PDAC development in response to hypoxia sensing.

The ability of ULK1/2 to sustain YAP-dependent PKM2 transcription under hypoxia and the exclusive downregulation of YAP protein in NE by ULK1/2 deficiency prompted us to explore the functional role of ULK1/2 in nuclear YAP stabilization. Although we did not observe major changes in YAP mRNA when ULK1, ULK2 or both was depleted by shRNA under hypoxia, depletion of ULK1, ULK2 or both greatly reduced accumulation of nuclear and total eYAP protein without affecting cytoplasmic eYAP abundance (Figure S2N), suggesting that ULK1/2 deficiency results in nuclear YAP degradation upon hypoxia. In echoing this notion, cycloheximide (CHX) pulse-chase experiments showed that the half-life of nuclear eYAP protein from ULK1, ULK2 or ULK1/2 shRNA-transfected cells were much shorter than that from Scr-transfected cells under hypoxia (Figure 3A). Nevertheless, the observed degradation of nuclear eYAP was abrogated when treating these cells with MG132, the 26S proteasome inhibitor (Figure 3B), indicating that nuclear YAP mainly undergoes proteasome-mediated degradation in the absence of ULK1/2 under hypoxia. Nuclear eYAP disappeared at a faster rate in SBI-pretreated cells with hypoxia stimuli when protein synthesis was inhibited by CHX (Figure S2O). As well, nuclear eYAP was substantially degraded after addition of SBI to the culture medium but restored to the levels as similar as seen before addition in the presence of MG132 (Figure S2P). Further cellular ubiquitination assay of the His-tagged Ub protein immobilized on Ni^2+^-nitrilotriacetic acid (Ni-NTA)-sepharose beads with an anti-YAP antibody depicted that ULK1, ULK2 or both depletion substantially increased YAP poly-ubiquitylation (poly-Ub) (Figure 3C), as judged by the elevated polyubiquitin conjugation of eYAP in the hypoxia-stimulated cells after ULK1, ULK2 or ULK1/2 shRNA transfection. Intriguingly, ectopic expression of Flag-ULK1/2 in the ULK1/2 shRNA-transfected cells with hypoxia stimuli partially mitigated the increased poly-Ub of eYAP resulting from ULK1/2 deficiency (Figure S3A). ULK1/2 deficiency is likely to attach the degradative Lys (K)48-linked polyubiquitin chains to YAP since eYAP purified from the hypoxia-stimulated cells lacking ULK1/2 was immunoblotted with a K48 linkage-specific instead of a K63 linkage-specific polyubiquitin antibody (Figure S3B and C). Collectively, these data propose that ULK1/2 stabilizes nuclear YAP by dismantling the degradative K48-linked polyubiquitin chains during hypoxia.

### ULK1 translocates into nucleus and phosphorylates YAP at Ser227 during hypoxia

We next sought to pursue the underlying mechanism of how ULK1/2 regulates nuclear YAP stabilization. For this purpose, we employed co-immunoprecipitation (co-IP) assay of eYAP in nuclear extractions from hypoxia-stimulated S-1 cells with an antibody against ULK1 to determine whether ULK1 could interact with YAP. We focused on ULK1 because its deficiency results in more nuclear eYAP turnover than ULK2 deficiency (Figure 3A), although ULK1 and ULK2 are highly homologous and possess almost identical substrate specificity. Figure 3D showed that the interaction of ULK1 with nuclear eYAP occurred only after hypoxia stimuli. Similar results were observed in the reciprocal co-IP assay of immunoprecipited ULK1 followed by western-blotting with an anti-YAP antibody showing that eYAP was detected in IPs of endogenous ULK1 protein under hypoxic conditions (Figure S3D). An *in vitro* protein binding assay with mixing purified His-tagged ULK1 or ULK2 and nuclear lysates from the hypoxia-stimulated cells expressing Flag-tagged wild-type YAP also revealed that the two proteins interacted directly (Figure 3E and S3E). Thus, ULK1 interacts with YAP in nucleus following hypoxia stimuli.

ULK1 phosphorylates numerous substrates preferentially through the consensus motif M/L-X-X-*S-φ (X represents any amino acid [aa], *denotes the phosphorylated residue and φ represents the hydrophobic aa) 26. Analysis of the YAP aa sequence identified a highly conserved ^224^MMN*SA^228^ peptide that exhibits optimal match with the ULK1 phosphorylation motif among human and other primates (Figure 3F), raising the possibility that ULK1 might phosphorylate YAP at Ser227. To address this proposal, we interrogate whether YAP could be phosphorylated by hypoxia and, if so, whether this is dependent on ULK1. WB analyses of the immunoprecipitated Flag from S-1 cells expressing Flag-tagged YAP with an antibody against phospho-Ser showed that hypoxia induced serine phosphorylation of YAP, but it was unable to do so when ULK1 had been depleted by shRNA (Figure 3G). Meanwhile, deactivation of ULK1/2 by SBI pretreatment blocked the hypoxia-stimulated YAP serine phosphorylation as efficiently as did ULK1 shRNA (Figure S3F). To directly clarify whether hypoxia evokes YAP phosphorylation at Ser227, we constructed phosphorylation site mutants of YAP S227A (in which Ser227 residue was mutated into a nonphosphorylatable alanine [A]) and S227E (in which Ser227 residue was mutated into a phosphorylation-mimetic glutamine [E]). Compared with wild-type YAP, mutant YAP S227A was refractory to the hypoxia-stimulated serine phosphorylation, whereas the mutant YAP S227E had high serine phosphorylation levels even in the absence of hypoxia (Figure 3H). The *in vitro* kinase assay with incubating purified His-tagged ULK1 and immunoprecipitates (IPs) of the Flag-tagged wild-type YAP or mutant YAP S227A further corroborated that ULK1 was able to phosphorylate wild-type YAP but not mutant YAP S227A (Figure 3I), verifying ULK1 phosphorylates YAP at Ser227 in response to hypoxia (Figure 3J).

**Figure 4:**
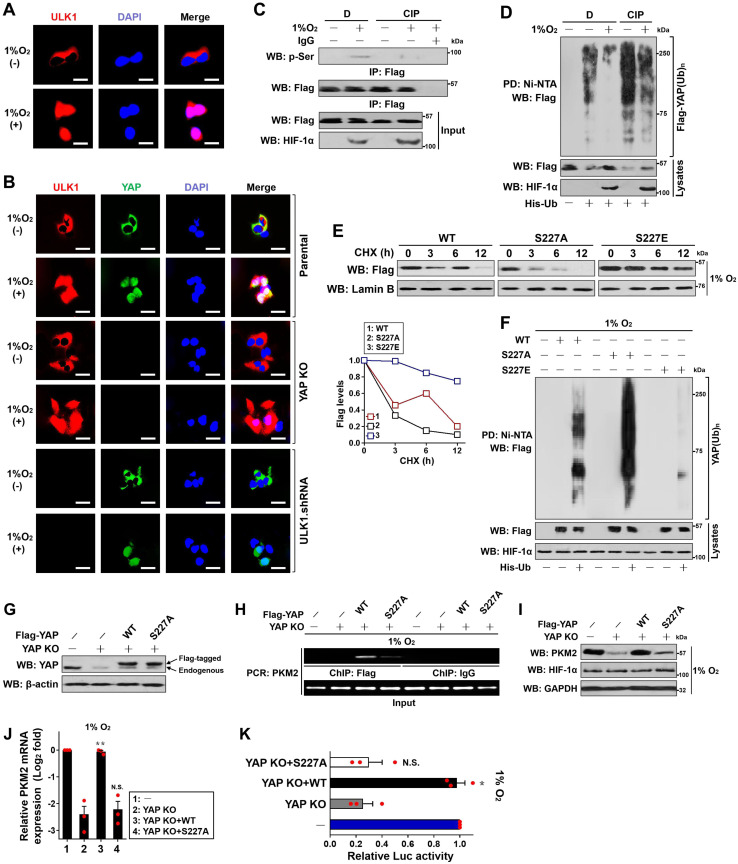
** Ser227 phosphorylation stabilizes YAP and augments PKM2 transcription upon hypoxia.** (A) Representative immunfluorescence images of ULK1 nuclear localization in SW-1990 cells before and after hypoxia stimuli. Scale bar = 20 μm. (B) Representative immunfluorescence images detecting the localization of ULK1 and YAP in YAP knockout (KO) or ULK1 shRNA transfected-SW-1990 cells before and after hypoxia stimuli, respectively. Scale bar = 20 μm. (C) Coimmunoprecipitation assay detecting the abundance of YAP Ser phosphorylation in Flag-tagged wild-type YAP-expressed SW-1990 cells with or without hypoxia stimuli in the presence of CIP treatment. (D) Cellular ubiquitination assays comparing the poly-Ub levels of YAP in Flag-tagged wild-type YAP-expressed SW-1990 cells with or without hypoxia stimuli in the presence of CIP treatment. (E) CHX pulse-chase experiments comparing the turnover of YAP protein in Flag-tagged wild-type YAP (WT)-, mutant YAP Ser227A (S227A)- and Ser227E (S227E)-expressed SW-1990 cells with hypoxia stimuli in the presence or absence of 20 μg/mL CHX treatment for the indicated times, respectively. (F) Cellular ubiquitination assays evaluating the poly-Ub levels of YAP protein in Flag-tagged wild-type YAP (WT)-, mutant YAP Ser227A (S227A)- and Ser227E (S227E)-expressed SW-1990 cells with hypoxia stimuli. (G) Western blotting examining the expression levels of YAP in YAP knockout (KO) SW-1990 cells with Flag-tagged wild-type YAP (WT) or mutant YAP Ser227A (S227A) reconstitution. (H) ChIP analysis for YAP binding to PKM2 gene promoter in YAP knockout (KO) SW-1990 cells with hypoxia stimulation in the presence of Flag-tagged wild-type YAP (WT) or mutant YAP Ser227A (S227A) reconstitution using the indicated antibodies. (I) Western-blotting analyses assessing the levels of PKM2 protein in YAP knockout (KO) SW-1990 cells with hypoxia stimulation in the presence of Flag-tagged wild-type YAP (WT) or mutant YAP Ser227A (S227A) reconstitution. (J) RT-qPCR analyses of PKM2 gene expression in YAP knockout (KO) SW-1990 cells with hypoxia stimulation in the presence of Flag-tagged wild-type YAP (WT) or mutant YAP Ser227A (S227A) reconstitution. Experiments were performed three times, each with quantitative RT-PCR in technical duplicate and real-time values were normalized to glyceraldehyde 3-phosphate dehydrogenase (GAPDH). Data are expressed as mean ± s.d. **P<0.01 versus YAP KO. Two-sided ANOVA with Bonferroni post hoc *t* test correction was used to calculate the *P* value. (K) Luciferase-reporter PKM2 promoter activity analysis of YAP knockout (KO) SW-1990 cells with hypoxia stimulation in the presence of Flag-tagged wild-type YAP (WT) or mutant YAP Ser227A (S227A) reconstitution. Data are expressed as mean ± s.d. of three independent experiments. *P<0.05 versus YAP KO. Two-sided ANOVA with Bonferroni post hoc *t* test correction was used to calculate the *P* value.

We noticed that the major portion of ULK1 within S-1 cells detected by both immunofluorescence staining and subcellular fractionation was presented in cytoplasm under normoxic conditions but predominantly accumulated in nucleus after hypoxia stimuli (Figure 4A and Figure S3G). Hypoxia induced nuclear accumulation of ULK1-KI to the equivalent extent as did of ULK1-WT (Figure S3H). The nuclear translocation of ULK1 was intact in eYAP KO cells following hypoxia stimuli while depletion of ULK1 blunted nuclear YAP accumulation under the same deoxygenated conditions (Figure 4B), presumably owing to the increased YAP turnover. These data, combined with the results obtained from co-IP analyses that ULK1 interacts with nuclear YAP and kinase assays that YAP undergoes the ULK1-induced phosphorylation at Ser227, suggest that ULK1 translocates into the nucleus and subsequently interacts with and phosphorylates YAP at Ser227 during hypoxia.

### Ser227 phosphorylation stabilizes YAP and augments PKM2 transcription upon hypoxia

Next, we investigated the significance of Ser227 phosphorylation in YAP stabilization. To approach this, we first tested whether stabilization of YAP occurs secondarily as a consequence of its serine phosphorylation during hypoxia. Indeed, treatment of the hypoxia-stimulated S-1 cells with an alkaline phosphatase (calf intestinal phosphatase [CIP]) abolished the serine phosphorylation of YAP as determined by WB analyses of immunoprecipitated Flag-YAP protein using the anti-phospho-Ser antibody (Figure 4C), which kinetically correlated with a higher poly-Ub conjugation and lower steady-state levels of YAP protein than the stimulated cells without treatment (Figure 4D), implicating that the hypoxia-stimulated serine phosphorylation dismantles poly-Ub of YAP and stabilizes it. CIP treatment also blocked the interaction of nuclear eYAP with HIF-1α triggered by ULK1 (Figure S2H). Upon hypoxia, introducing the phospho-defective S227A mutant remarkably accelerated turnover of YAP in the presence of CHX treatment, while the mutant YAP S227E, which by itself mimics well the ULK1-phosphorylated status under hypoxic conditions, seems to be more stable than the wild-type YAP (Figure 4E). In line with the inhibitory role of Ser227 phosphorylation in YAP turnover, mutant YAP S227A underwent much more polyubiquitylation than did wild-type YAP, whereas the mutant YAP S227E was resistant to polyubiquitylation during hypoxia (Figure 4F), supporting the notion that the hypoxia-stimulated YAP Ser227 phosphorylation counteracts the polyubiquitylation-mediated YAP degradation. Unlike wild-type YAP, the mutant YAP S227A rarely interacted with HIF-1α even in the presence of ULK1 (Figure S2H).

Take into account that YAP is required for the hypoxia-dependent, ULK1/2-mediated PKM2 transcription, we wondered whether YAP Ser227 phosphorylation, which ultimately leads to YAP stabilization, influences PKM2 transcription under hypoxia. To this end, we reconstituted the eYAP KO cells with the Flag-tagged wild-type YAP and mutant YAP S227A, respectively (Figure 4G). As measured by ChIP assay with an anti-Flag antibody, the mutant YAP S227A, which becomes unstable following hypoxia stimuli, had a much-impaired ability to occupy at *PKM2* gene promoter in comparison with wild-type YAP (Figure 4H). In addition, eYAP KO markedly downregulated protein expression (Figure 4I), mRNA levels (Figure 4J) and promoter activity (Figure 4K) of PKM2 under hypoxia, but these effects were all reversed by reconstitution of the wild-type YAP (*P* < 0.01 and *P* < 0.05) rather than mutant YAP S227A. These data indicate that the Ser227 phosphorylation-dependent YAP stabilization transcriptionally upregulates PKM2 under hypoxic circumstances.

### ULK1/2-YAP axis contributes to hypoxic glycolysis and tumorigenesis of PDAC cells

**Figure 5:**
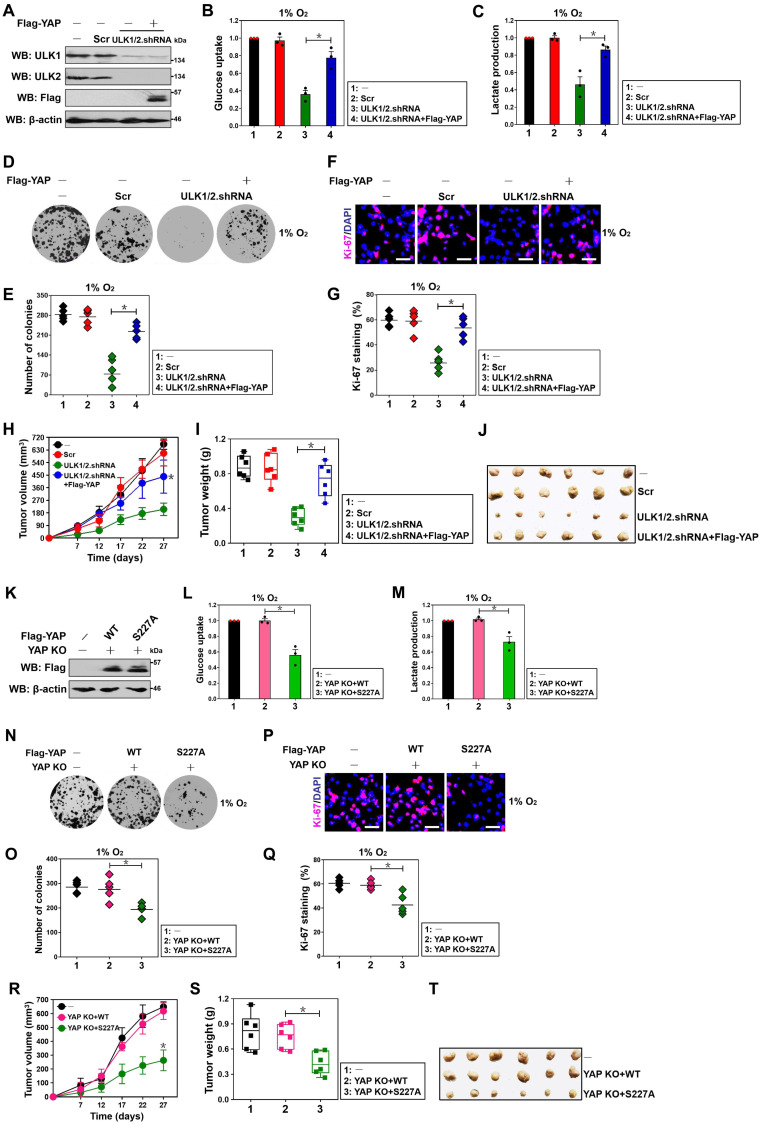
** ULK1/2-YAP axis contributes to hypoxic glycolysis and tumorigenesis of PDAC cells.** (A) Western-blotting analyses detecting the levels of ULK1, ULK2 and Flag protein in ULK1/2 shRNA-transfected SW-1990 cells with or without Flag-tagged wild-type YAP expression. (B and C) Glucose consumption (B) or lactate production (C) of ULK1/2 shRNA-transfected SW-1990 cells with or without Flag-tagged wild-type YAP expression under hypoxia. Data are expressed as mean ± s.d. of three independent experiments. *P<0.05. Two-sided ANOVA with Bonferroni post hoc *t* test correction was used to calculate the *P* value. (D-G) Colony formation assays (D and E) and Ki-67 staining analyses (F and G) of ULK1/2 shRNA-transfected SW-1990 cells with or without Flag-tagged wild-type YAP expression under hypoxia. Data are expressed as mean ± s.d. of five independent experiments. *P<0.05. Two-sided ANOVA with Bonferroni post hoc *t* test correction was used to calculate the *P* value. Scale bar = 100 μm. (H-J) Tumor volume (H), weight (I) and representative images (J) of xenografts excised from the tumor-bearing mice. The ULK1/2 shRNA-transfected SW-1990 cells (1×10^7^) with or without Flag-tagged wild-type YAP expression were subcutaneously inoculated into the right flank of nude mice (n=6). The mice were sacrificed and the tumors were excised and measured on day 27. Data are presented as mean ± s.d. *P<0.05 versus ULK1/2.shRNA. Two-sided ANOVA with Bonferroni post hoc *t* test correction was used to calculate the *P* value. (K) Western-blotting analyses determining the levels of Flag protein in YAP knockout (KO) SW-1990 cells with Flag-tagged wild-type YAP (WT) or mutant YAP Ser227A (S227A) reconstitution. (L and M) Glucose consumption (L) or lactate production (M) of YAP knockout (KO) SW-1990 cells with Flag-tagged wild-type YAP (WT) or mutant YAP Ser227A (S227A) reconstitution under hypoxia. Data are expressed as mean ± s.d. of three independent experiments. *P<0.05. Two-sided ANOVA with Bonferroni post hoc *t* test correction was used to calculate the *P* value. (N-Q) Colony formation assays (N and O) and Ki-67 staining analyses (P and Q) of YAP knockout (KO) SW-1990 cells with Flag-tagged wild-type YAP (WT) or mutant YAP Ser227A (S227A) reconstitution under hypoxia. Data are expressed as mean ± s.d. of five independent experiments. *P<0.05. Two-sided ANOVA with Bonferroni post hoc *t* test correction was used to calculate the *P* value. Scale bar = 100 μm. (R-T) Tumor volume (R), weight (S) and representative images (T) of xenografts excised from the tumor-bearing mice. The YAP knockout (KO) SW-1990 cells (1×10^7^) with Flag-tagged wild-type YAP (WT) or mutant YAP Ser227A (S227A) reconstitution were subcutaneously inoculated into the right flank of nude mice (n=6). The mice were sacrificed and the tumors were excised and measured on day 27. Data are presented as mean ± s.d. *P<0.05 versus YAP KO+WT. Two-sided ANOVA with Bonferroni post hoc *t* test correction was used to calculate the *P* value.

To understand whether YAP is involved in the ULK1/2-dependent hypoxic glycolysis, we transfected the ULK1/2 shRNA-transfected S-1 cells with Flag-tagged wild-type YAP (Figure 5A) and then stimulated them with hypoxia. Notably and consistent with the results described for PKM2 transcription inhibition after genetic depletion of ULK1/2, the ULK1/2 shRNA-transfected cells had lower magnitude of glucose uptake and lactate production than the Scr-transfected cells in the presence of hypoxia, which could be abrogated by concurrent expression of YAP (*P* < 0.05, Figure 5B and C). Examination of clonogenic outgrowth using colony formation assays from the ULK1/2 shRNA-transfected, but not the ULK1/2 shRNA plus Flag-YAP-cotransfected S-1 cells identified a great reduction in the number and size of surviving colonies when compared with the parental or the Scr-transfected cells under hypoxia (*P* < 0.05, Figure 5D and E). In agreement with these observations, the percentage of Ki-67 staining, an indicative of cell proliferation, was significantly decreased in the ULK1/2 shRNA-transfected cells but was restored after concomitant expression of Flag-YAP (*P* < 0.05, Figure 5F and G). Coincidently, overexpression of exogenous YAP was sufficient to rescue the decreased glucose uptake, lactate production, clonogenic outgrowth and cell proliferation in the SBI-administered cells under hypoxia (*P* < 0.001 and *P* < 0.05, Figure S4A-D). We also enrolled the subcutaneous xenograft experiments to investigate the behavior of ULK1/2-YAP axis in tumorigenicity of PDAC cells *in vivo*. In this analysis, we observed that tumors arised from nude mice inoculated with cells bearing ULK1/2 shRNA grew more slowly, had milder weight and contained less PKM2 staining than those arised from mice inoculated with cells bearing Scr, while the tumors arised from nude mice inoculated with cells bearing ULK1/2 shRNA plus Flag-YAP did not (*P* < 0.05, Figure 5H-J and Figure S4E). Together with the aforementioned data, these results indicate that ULK1/2-YAP axis contributes to the hypoxic glycolysis and tumorigenesis of PDAC cells.

Along with the contributing role of YAP Ser227 phosphorylation in PKM2 transcription, the eYAP KO cells reconstituted with mutant YAP S227A had decreased magnitude of glucose uptake and lactate production in contrast to the KO cells reconstituted with wild-type YAP during hypoxia, which had comparable magnitude of glucose uptake and lactate production with the control cells (*P* < 0.05, Figure 5K-M). As estimated by colony formation assays, Ki-67 staining analyses and shown in Figure 5N-Q, clonogenic outgrowth and Ki-67 staining of eYAP KO cells with hypoxia stimuli were apparently impeded after mutant YAP S227A reconstitution instead of wild-type YAP reconstitution (*P* < 0.05). Additional evidence that YAP Ser227 phosphorylation facilitates PDAC tumorigenesis was delineated by the subcutaneous xenograft experiments showing that tumors derived from mice injected with the eYAP KO cells harboring mutant YAP S227A displayed statistically significant slower growth rate and smaller mass than those derived from mice injected with the KO cells harboring wild-type YAP (*P* < 0.05, Figure 5R-T). Further histopathological analysis demonstrated that the subcutaneously inoculated eYAP KO tumors with mutant YAP S227A reconstitution presented weaker PKM2 staining than did the inoculated KO tumors with wild-type YAP reconstitution (Figure S4F). Collectively, these data implicate that YAP Ser227 phosphorylation is necessary for PDAC cells to maintain hypoxic glycolysis and growth under hypoxic circumstances *in vitro* as well as develop tumors in mice.

### Correlation between ULK1/2, YAP and PKM2 in PDAC

**Figure 6:**
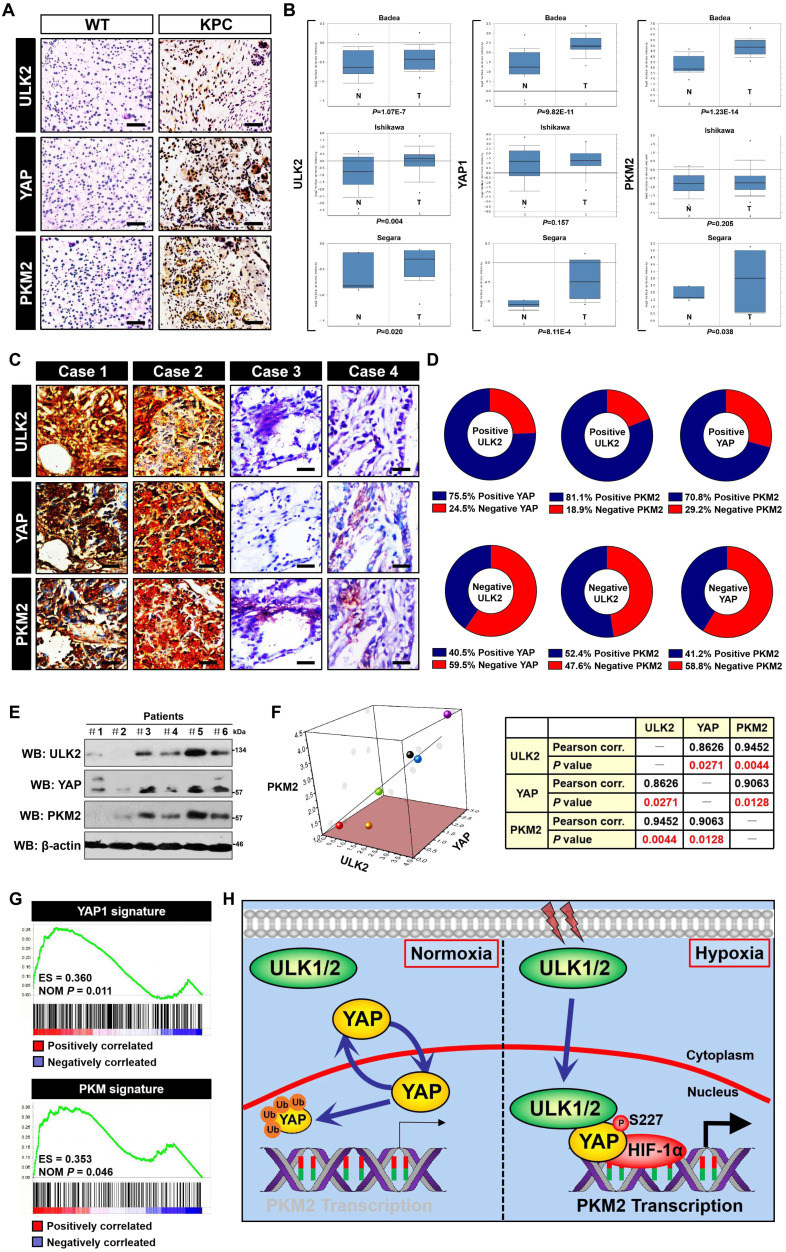
**Correlation between ULK1/2, YAP and PKM2 in PDAC.** (A) Representative images of immunohistochemistry comparing expression of ULK2, YAP and PKM2 in pancreas tissues from the wild-type mice and PDAC tissues from the KPC (*Pdx1*-Cre; LSL-Kras^G12D/+^; Trp53^fl/+^) mice. Scale bar = 100 μm. (B) Comparison of ULK2, YAP1 and PKM2 mRNA levels between normal pancreatic tissues and PDAC tumor samples from three independent cohorts in the Oncomine database. (C and D) Representative cases stained by immunohistochemistry (C) and pie charts (D) showing expression of ULK2, YAP and PKM2 in 95 primary human PDAC specimens are positively correlated with each other. Scale bar = 50 μm. (E and F) Western blotting analyses of ULK2, YAP and PKM2 protein correlation in 6 freshly collected human PDAC specimens. The *P* value shown was calculated by Pearson's correlations. (G) Gene set enrichment analysis (GSEA) plot showing the correlation of YAP1 and PKM signature with ULK2 in GSE55643, respectively. (H) Schematic model proposing the nuclear localization of ULK1/2 within PDAC cells under hypoxic microenvironment and its role in YAP Ser227 phosphorylation, stabilization and subsequent transcriptional coactivation of PKM2 together with HIF-1α.

To gain insight into the relevance of ULK1/2,YAP and PKM2 in PDAC, levels of ULK2,YAP and PKM2 protein expression in tumour tissues from KPC (*Pdx1*-Cre; LSL-Kras^G12D/+^; Trp53^fl/+^) mice were examined by immunohistochemical staining, which revealed that ULK2,YAP and PKM2 were strongly expressed in KPC tumour tissues (*P* < 0.0001, Figure 6A and Figure S5A). By contrast, normal pancreas tissues from the wild-type littermates exhibited the opposite relationship. Analyses of both ULK1 and ULK2 mRNA expression in matched normal and PDAC tissues from three independent cohorts (Badea [n = 78], Ishikawa [n = 49] and Segara [n = 17]) using the Oncomine database (Figure 6B) revealed that ULK2 was overexpressed in PDAC tumor tissues as compared to normal adjacent pancreas tissues (*P =* 1.07E-7 for Badea,* P =* 0.004 for Ishikawa and* P* = 0.02 for Segara, respectively), in concert with the levels of YAP1 (*P =* 9.82E-11 for Badea,* P =* 0.157 for Ishikawa and* P* = 8.11E-4 for Segara, respectively) and PKM2 (*P =* 1.23E-14 for Badea,* P =* 0.205 for Ishikawa and* P* = 0.038 for Segara, respectively), but ULK1 mRNA expression in PDAC tissues did not differ significantly from those in normal pancreas tissues among these three cohorts (*P =* 0.993 for Badea,* P =* 0.837 for Ishikawa and* P* = 1.000 for Segara, respectively; data not shown). The upregulation of ULK2, YAP1 and PKM2 mRNA in PDAC tissues were further validated by RNA-sequencing data in a GSE15471 cohort with 36 human PDAC and matching normal pancreas specimens from NCBI's Gene Expression Omnibus (GEO) website showing that ULK2 (t = 5.731, 95% CI = 0.44 to 0.91,* P* < 0.0001), YAP1 (t = 7.685, 95% CI = 0.94 to 1.60,* P* < 0.0001) and PKM2 (t = 9.572, 95% CI = 1.22 to 1.85,* P* < 0.0001) levels were recurrently higher in PDAC samples than in normal pancreas samples (Figure S5B). Inspection of cancer genomics data from a combined study of 1034 pancreatic cancer samples in the Cancer Genome Atlas (TCGA) and International Cancer Genome Consortium (ICGC) data sets using cBioPortal depicted that ULK1/2 is predominantly mutated or amplified in the tumour samples with high expression and mutation of YAP1 or PKM (Figure S5C). In addition, survival analyses of 177 pancreatic adenocarcinoma patient from TCGA datasets demonstrated that patients with the combination of high ULK1/2, YAP1 and PKM signature displayed shorter overall survival than those with low ULK1/2, YAP1 and PKM signature (Log-rank test,* P* = 2.3e-05 and *P* = 1e-05, Figure S5D), implying that both transcriptomic and genomic upregulation of these three genes may reflect PDAC tumorigenesis.

To dissect the pathologic relationship of the findings obtained in KPC mice and bioinformatic studies, we analyzed 95 tumor tissues from clinical patients diagnosed with PDAC for immunohistochemical staining. We quantified ULK2, YAP and PKM2 expression in these samples and identified 55.8% (53 out of 95) samples that were positive for ULK2 as well as 44.2% (42 out of 95) samples that were negative for ULK2, indicating that ULK2 might be hyperactivated in the majority of patients with PDAC. Of note, 24.5% samples with positive ULK2 expression exhibited low levels of YAP (13 cases), while 40.5% samples with negative ULK2 expression showed high expression of YAP (17 cases) (*r* = 0.332, *P* = 0.001; Figure 6C, D and Figure S5E). Moreover, the positive ULK2 was detected in 81.1% specimens with strong expression of PKM2 and in 18.9% specimens with weak expression of PKM2, while the negative ULK2 was detected in 52.4% specimens with strong expression of PKM2 and 47.6% specimens with weak expression of PKM2 (*r* = 0.307, *P* = 0.002; Figure 6C, D and Figure S5E). WB analyses of snap-frozen tumor tissues from 6 clinical PDAC patients who had undergone radical resection showed that ULK2 protein expression was positively correlated with the levels of YAP (*P* = 0.0271) and PKM2 (*P* = 0.0044) (Figure 6E and F). We integrated YAP1 and PKM coexpression genes in the Cancer Cell Line Encyclopedia (CCLE) dataset and separately defined them as the YAP1 and PKM signature. By performing gene set enrichment analysis (GSEA) in published PDAC expression profiles, we observed a positive correlation of YAP1 and PKM signature with ULK2 (*P* = 0.011 and *P* = 0.046; Figure 6G), respectively, demonstrating high concordance with ULK2 in terms of involved pathways from gene ontology (GO) analyses (Figure S5F). These data suggest that ULK1/2, YAP and PKM2 are correlated with each other in human PDAC.

### Targeting ULK1/2 potentiates therapeutic efficacy of 2-DG and 3-BP against PDAC

**Figure 7:**
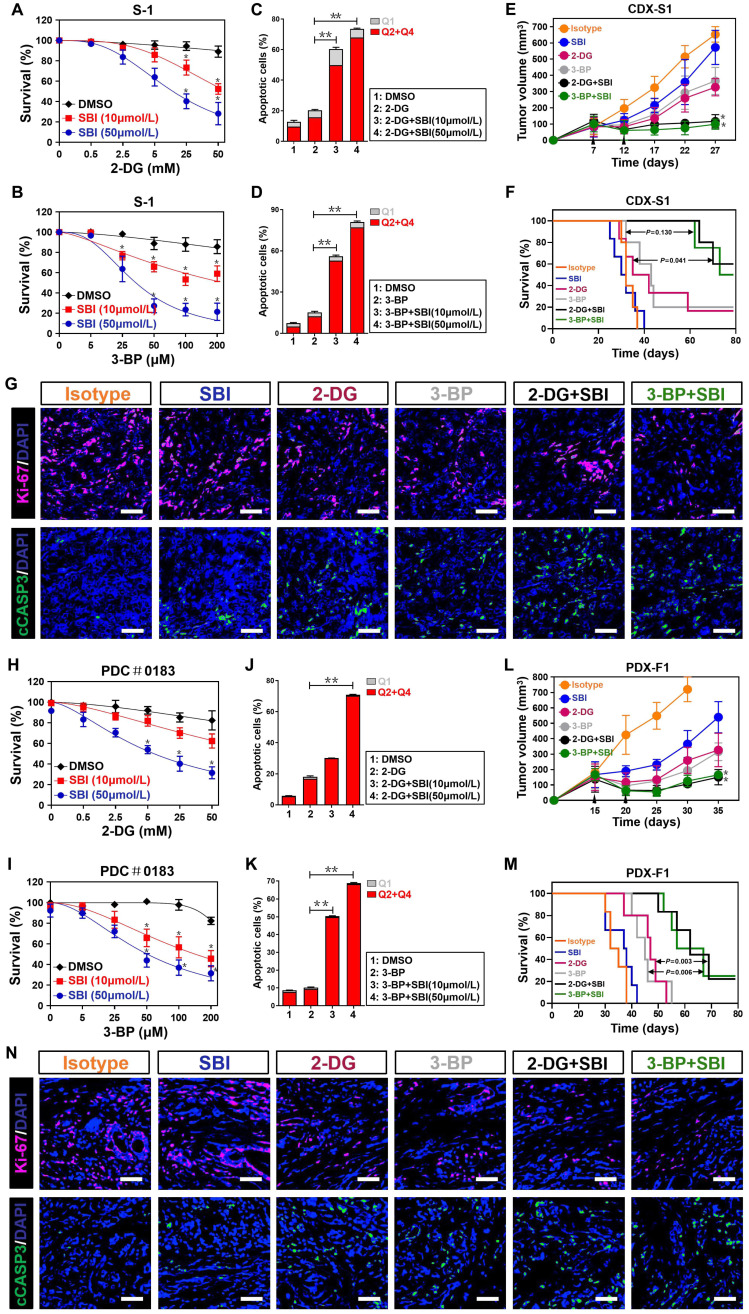
** Targeting ULK1/2 potentiates therapeutic efficacy of 2-DG and 3-BP against PDAC.** (A and B) MTT assays meauring cell viability of SW-1990 cells treated with the indicated concentrations of 2-DG (A) and 3-BP (B) with or without SBI-0206965 (SBI) administration. Data are expressed as mean ± s.d. of three independent experiments. *P<0.05 versus DMSO. Two-sided ANOVA with Bonferroni post hoc *t* test correction was used to calculate the *P* value. (C and D) Quantification of flow cytometry with Annexin-V/PI staining in SW-1990 cells treated with 25 mM 2-DG (C) and 100 μM 3-BP (D) with or without SBI-0206965 (SBI) administration. Data are expressed as mean ± s.d. of three independent experiments. *P<0.05 and **P<0.01. Two-sided ANOVA with Bonferroni post hoc *t* test correction was used to calculate the *P* value. (E) Tumor volume of xenografts from the tumor-bearing mice. SW-1990 cells (S1, 1×10^7^) were subcutaneously inoculated into the right flank of nude mice. 2-DG or 3-BP in combination with or without SBI-0206965 (SBI) were intraperitoneally injected into nude mice for 5 day-intervals from seven days after inoculation. Data are presented as mean ± s.d. *P<0.05 versus 2-DG or 3-BP. Two-sided ANOVA with Bonferroni post hoc *t* test correction was used to calculate the *P* value. CDX, cell-derived xenograft. (F) Overall survival of mice with S1 xenografts treated with 2-DG or 3-BP in combination with or without SBI-0206965 (SBI). Log-rank test was used to calculate the *P* value. (G) Representative immunfluorescence images for Ki-67 (red) and cleaved caspase-3 (cCASP3, green) staining for xenografts as in (I and J). Scale bar = 100 μm. (H and I) MTT assays meauring cell viability of PDC treated with the indicated concentrations of 2-DG (H) and 3-BP (I) with or without SBI-0206965 (SBI) administration. Data are expressed as mean ± s.d. of three independent experiments. *P<0.05 versus DMSO. Two-sided ANOVA with Bonferroni post hoc *t* test correction was used to calculate the *P* value. (J and K) Quantification of flow cytometry with Annexin-V/PI staining in PDC treated with 25 mM 2-DG (J) and 100 μM 3-BP (K) with or without SBI-0206965 (SBI) administration. Data are expressed as mean ± s.d. of three independent experiments. **P<0.01. Two-sided ANOVA with Bonferroni post hoc *t* test correction was used to calculate the *P* value. (L) Tumor volume of xenografts from the tumor-bearing mice. Small pieces of tumor specimens from PDAC patient were mixed with matrigel and subcutaneously inoculated into the right flank of nude mice. 2-DG or 3-BP in combination with or without SBI-0206965 (SBI) were intraperitoneally injected into nude mice for 5 day-intervals from fifteen days after inoculation. Data are presented as mean ± s.d. *P<0.05 versus 2-DG or 3-BP. Two-sided ANOVA with Bonferroni post hoc *t* test correction was used to calculate the *P* value. PDX, patient-derived xenograft. (M) Overall survival of mice with PDX xenografts treated with 2-DG or 3-BP in combination with or without SBI-0206965 (SBI). Log-rank test was used to calculate the *P* value. (N) Representative immunfluorescence images for Ki-67 (red) and cleaved caspase-3 (cCASP3, green) staining for xenografts as in (L and M). Scale bar = 100 μm.

It has been reported that low levels of glycolysis in cancer cells are necessary for the therapeutic response of hexokinase I inhibitor 2-deoxyglucose (2-DG) and hexokinase II inhibitor 3-bromopyruvate (3-BP) 32, 33. Given our above-stated data demonstrate that deactivation of ULK1/2 by SBI impairs glycolysis, we speculated whether SBI would render PDAC cells more sensitive to 2-DG and 3-BP. To this end, we treated S-1 cells with 2-DG and 3-BP in the presence or absence of SBI, respectively. Cell viability curves, as measured by MTT assay, showed that either 2-DG or 3-BP treatment alone caused a limited degree of reduction in cell survival, but such effects became prominent after SBI combination (*P* < 0.05; Figure 7A and B). When compared with cells with 2-DG or 3-BP single treatment, cells cotreated with 2-DG or 3-BP plus SBI had higher percentage of apoptosis in the flow cytometry (FCM) analyses with Annexin V-FITC/PI staining (*P* < 0.01; Figure 7C, D and Figure S6A, B). To determine the efficacy of combined ULK1/2 blockade with 2-DG or 3-BP *in vivo*, mice bearing S-1 xenografts were administered 2-DG or 3-BP plus SBI during the course of therapy. We observed a decreased tumour volume and improved survival in mice receiving combination therapy as compared with those receiving 2-DG or 3-BP therapy alone, respectively (*P* < 0.05 and *P* = 0.041; Figure 7E, F and S6C). Reduction in Ki-67 staining and augment in the number of cleaved caspase-3-positive cells, were observed in the tumors deriving from mice treated with 2-DG or 3-BP plus SBI in comparison to those with 2-DG or 3-BP single treatment (Figure 7G).

To further validate our data in a cellular model that mimicking well the anti-neoplastic aspects of clinical patients, we tested the efficacy of ULK1/2 blockade combined with 2-DG or 3-BP using a pancreatic patient-derived cancer cell (PDC). As expected, 2-DG or 3-BP more effectively killed PDC in the presence of SBI (*P* < 0.05 and *P* < 0.01; Figure 7H-K and Figure S6D, E). The impaired tumor growth, improved mice survival, decreased Ki-67 staining and increased number of cleaved caspase-3-positive cells were also observed from the patient-derived xenograft (PDX) model that better reflects tumor heterogeneity (*P* < 0.05, *P* = 0.003 and *P* = 0.006; Figure 7L-N and S6F). However, exposure to these treatment regimen did not lead to remarkable alterations in body weight, platelet (PLT) count, alanine aminotransferase (ALT) and serum creatinine (Scr) levels of mice, respectively (Figure S6G-N), excluding any overt signs of toxicity. Therefore, targeting ULK1/2 in combination with 2-DG or 3-BP synergistically provides better therapeutic efficacy against PDAC.

## Discussion

In order to adapt to and even benefit from hypoxic tumor microenvironment, cancer cells often reprogram their carbon metabolism to a state favorable for maintaining distinct malignant traits. Elevated glycolysis resulting from aberrant amplification of PKM2 gene in cancer cells is accompanied by the onset of hypoxia sensing, which simultaneously instigates autophagic flux through activation of ULK1/2 34, 35, making the two catabolic events central step for tumorigenesis individually. While both autophagy and glycolysis are known to be important for hypoxia sensing, our understanding of how autophagy intersects with glycolysis to orchestrate malignant behavior is incomplete and the molecular mechanism underlying their crosstalk in response to hypoxia sensing remains largely unknown. Our recent study demonstrated that the nuclear YAP cooperates with HIF-1α to transactivate PKM2, accelerate glycolysis and promote PDAC development upon hypoxia 10. Based on these premises and evidences, we postulate that ULK1/2 might function as a “redox sensor” to integrate oncogenic signaling and fuel availability for PDAC development via modulating the YAP-driven glycolysis.

To our knowledge, we believe the current study is of particular interest to unveil that: (1) ULK1/2, but not ULK3, play a pivotal role in the hypoxic glycolysis mediated by PKM2 independent of BNIP3. Mechanistically, hypoxia stimulates ULK1 to translocate into nucleus, where ULK1 directly interacts with and phosphorylates YAP at Ser227, thereby stabilizing YAP through blockade of ubiquitin-proteasome system (UPS) and facilitating YAP recruitment at *PKM2* gene promoter to augment PKM2 transcription together with HIF-1α (Figure 6H). (2) Pharmacological deactivation of ULK1/2 potentiates the anti-neoplastic efficacy of 2-DG and 3-BP in cellular experiments *in vitro*, CDX and PDX models of PDAC *in vivo*, fitting with our proposal that targeting ULK1/2 perturbs PKM2 transcription as a result of nuclear YAP turnover, which led to low levels of intrinsic glycolysis, thus enhancing vulnerability and responsiveness of PDAC to 2-DG and 3-BP. Our future work will aim at ascertaining the exact mechanism of how hypoxia evokes nuclear translocation of ULK1 and pinpointing whether the interdependence between ULK1/2 and YAP-driven glycolysis with respect to hypoxia sensing is a common feature in other solid tumors.

Our study unearth a principal role of ULK1/2 kinase activity in the YAP-driven glycolysis mediated by PKM2 under hypoxia. Futher biochemical analyses suggest that depletion of ULK1/2, but not that of ULK3, downregulates the hypoxia-dependent PKM2 transcription, the effect that could be abrogated by YAP knockout. In this regard, ULK1/2 may act as an oncogenic intermediate for the YAP-dependent PKM2 transcription and glycolysis in response to hypoxia sensing. Although the underlying mechanism remain unclear, somatic mutation and overexpression of ULK1/2 has been observed in several solid tumors such as colon cancer, hepatocellular carcinoma (HCC) and breast cancer 36-38. Given mutant gene or protein is usually amplified during tumor development, it may explain why PDAC cells lacking ULK1/2, as mentioned here in our study, had impaired tumorigenic phenotypes both *in vitro* and *in vivo*. In addition, our data show that ULK1/2 expression are positively correlated with YAP and PKM2 levels in tumor tissues from KPC mice and samples from clinical patients with PDAC. These findings renovate the concept that the increased mutant ULK1/2 expression might be a possible mechanism responsible for the elevated YAP and PKM2 levels in human PDAC. We did not observe significant increase in glycolysis of the ULK1/2-null PL45 cells following restoration of ULK1 expression under normoxic conditions, suggesting that ULK1/2 on their own may not be the primary factor for glycolysis of PDAC, whose carbon metabolism is reprogrammed after being exposed to a permissive microenvironment.

Previous studies have shown that YAP is a nucleocytoplasmic shuttling protein with labile characterization. Large tumor suppressor gene 1/2 (LATS1/2) phosphorylates cytoplasmic YAP, leading to secondary phosphorylation of YAP by casein kinase 1δ/ε, which recruits β-transducin repeat-containing protein (β-TrCP) for YAP turnover 39. SCF^Fbxw7α^ is identified as a bona fide E3 ligase of YAP and its deficiency enhances steady-levels of YAP protein in cancer cells 40. YAP is thought to be stabilized by nuclear inclusion of mitogen-activated protein kinase kinase (MEK) protein 41. In our search, we discover that the ULK1/2-dependent Ser227 phosphorylation is essential for nuclear YAP stabilization because depletion of ULK1/2, especially ULK1, which abolishes Ser227 phosphorylation of nuclear YAP under hypoxia, blocks nuclear YAP turnover through dismantling the K48-linked poly-ubiquitylation. Intriguingly, mutation of Ser227 residue into nonphosphorylatable alanine, accelerates turnover of nuclear YAP during hypoxia, parallel to that caused by ULK1/2 depletion. Further biochemical studies are needed to determine whether the ULK1/2-dependent YAP Ser227 phosphorylation occurs in other organelles where YAP exerts its oncogenic functions 42, 43.

Our study identify ULK1 as an upstream kinase that phosphorylates nuclear YAP at Ser227. ULK homologs seem to be predominantly cytoplasmic in resting cells 44. It is worth noting that after hypoxia stimuli, a major fraction of ULK1 translocates to nucleus, which is important for the formation of ULK1-YAP immunocomplex. We do not know the precise mechanism of how hypoxia regulates the subcellular distribution of ULK1 in PDAC cells hitherto. Catalytic activity of ULK1 is controlled by its autophosphorylation at Ser757, which allows ULK1 to disassociate from adenosine monophosphate (AMP)-activated protein kinase (AMPK) 45. Neither do we know the relationship between Ser757 phosphorylation of ULK1 and its nuclear translocation in response to hypoxia. Take into account that HIF-1α participates in hypoxia-stimulated autophagy as a putative manner for adaption and cell survival 46, 47, future studies are warranted to illustrate whether HIF-1α transactivation is a prerequisite for nuclear ULK1 localization and subsequent YAP Ser227 phosphorylation under hypoxia.

Several post-translational modifications (PTMs) have been shown to influence the transcriptional activity of YAP in the development and progression of human malignancies. Mono-methylation of nuclear YAP at K342 by SET domain containing 1A (SET1A) results in YAP transactivation and promotes tumorigenesis 48. The sirtuin 1 (SIRT1)-triggered deacetylation enhances transcriptional activity of YAP and confers chemoresistance 49. Our work indicate that the ULK1-inducible Ser227 phosphorylation is instrumental for nuclear YAP stabilization and subsequent PKM2 transcription upon hypoxia. This not only unravels a regulatory role of autophosphorylation in YAP stabilization and PKM2 transcription but also provide a rationale to explain why mutant YAP S227A is sufficient to compromise the ability of PDAC cells to uptake glucose and produce lactate upon hypoxia sensing. Since YAP is a key coactivator in determination of cancer cell fate, such molecular mechanism necessitates the participation of multiple oncogenic events to coordinate with ULK1/2 for the fine tuning of YAP function under pathological circumstances.

ULK1/2 has been linked to diverse oncogenic or tumor-suppressive signalling cascades (e.g., KRAS^G12D^/Copper, PI3K/AKT/mTOR, FAK/RhoA, STK11/liver kinase-B1 [LKB1]) that are engaged in pathogenesis of solid tumors from various types 50-52. Interestingly, at least some of these pathways, such as KRAS^G12D^/copper and STK11/LKB1, regulate ULK1/2 kinase activity, favoring a reciprocal model in which autophagosome formation and oncogenic signalling transduction act together. More importantly, evidence clearly points to a convergent role of Copper in autophagy of cancer cells 53, 54, implying a functional association and connection between ULK1/2 and the Copper complex. It is conceivable that ULK1/2, through being incorporated into and interacting with Copper, is activated by KRAS^G12D^ to exert distinct tumorigenic functions in PDAC.

The role of autophagy in PDAC immunity has been less well studied than in PDAC pathogenesis. Beside having one of the highest PD-L1 expression among solid tumors, PDAC shows immunosuppressive phenotypes with low levels of infiltrating T-cells and impaired antigen presentation 55. Recent studies illustrate that tumors with hyperactivated autophagy predict poor response to anti-PD-1 therapy, supporting the concept that further benefits and outcomes might be acquired from addition of autophagy inhibitors to anti-PD-1 therapy 56. These evidences and our studies together suggest that the oncogenic activity of ULK1/2 might serve as the autophagy initiator and immunosuppressor to perpetuate PDAC development.

Our work, however, have some limitations. First, the functional and mechanistic data in current study are almost obtained from cellular and biochemical assays *in vitro* as well as subcutaneous xenograft experiments *in vivo*, it would be necessary to perform studies to validate whether ULK1/2 deficiency is able to blunt YAP stabilization, PKM2 transcription, glycolysis and PDAC development using ULK1/2-conditional knockout mouse model. Second, yet our data identify ULK1 as the upstream kinase for nuclear YAP stabilization in PDAC cells, the possibility that other Ser/Thr kinases might also be involved in such process can not be ruled out.

In summary, our findings reveal a previously unappreciated crosstalk between ULK1/2-initiated autophagy and glycolysis in PDAC development under hypoxic microenvironment via stabilization of nuclear YAP, which serves as a niche for malignant growth and drug resistance 57. Of note, PDAC therapy could further benefit from combination of small-molecule ULK1/2 kinase inhibitor and 2-DG or 3-BP, supporting investigation of autophagy blockade in combination with the two anti-cancer agents as a promising therapeutic approach in clinical trials of PDAC patients.

## 2772 Supplementary Material

Supplementary figures.
