# Supplementary material for: Crosstalk between hypoxia-sensing ULK1/2 and YAP-driven glycolysis fuels pancreatic ductal adenocarcinoma development
Source: Int J Biol Sci. 2021 Jul 5;17(11):2772–94. doi: 10.7150/ijbs.60018 (PMC8326115; doi:10.7150/ijbs.60018)
Supplement: Supplementary file 1 — Supplementary figures. [file ijbsv17p2772s1.pdf]

Crosstalk between hypoxia-sensing ULK1/2 and YAP-driven glycolysis fuels pancreatic ductal adenocarcinoma development

Yu Jia[1], Hui-Yan Li[2], Ying Wang[3], Jue Wang[4], Jing-Wen Zhu[2], Yan-Yan Wei[2], Lu Lou[2], Xing Chen[2], Shi-Jing Mo[2*]

[1]*Cancer Research Center, Tongji Hospital, Tongji Medical College, Huazhong University of Science and Technology, Wuhan 430030, Hubei, P.R.China*

[2]*General Surgical Laboratory, The First Affiliated Hospital, Sun Yat-Sen University, Guangzhou, 510080, Guangdong, P.R.China*

[3]*Department of Surgery, Huashan Hospital, Fudan University, Shanghai 200040, P.R.China*

[4]*Department of Pathology, The First Affiliated Hospital, Sun Yet-Sen University, Guangzhou 510080, Guangdong, P.R.China*

Corresponding author:

[*]Shi-Jing Mo, MD, PhD

General Surgical Laboratory

The First Affiliated Hospital

Sun Yat-Sen University

Guangzhou, 510080, Guangdong, P.R.China

Email: moshij@mail2.sysu.edu.cn

ORCID: https://orcid.org/0000-0002-2537-3255

Supplemental Figures 1-6 and Figure legends

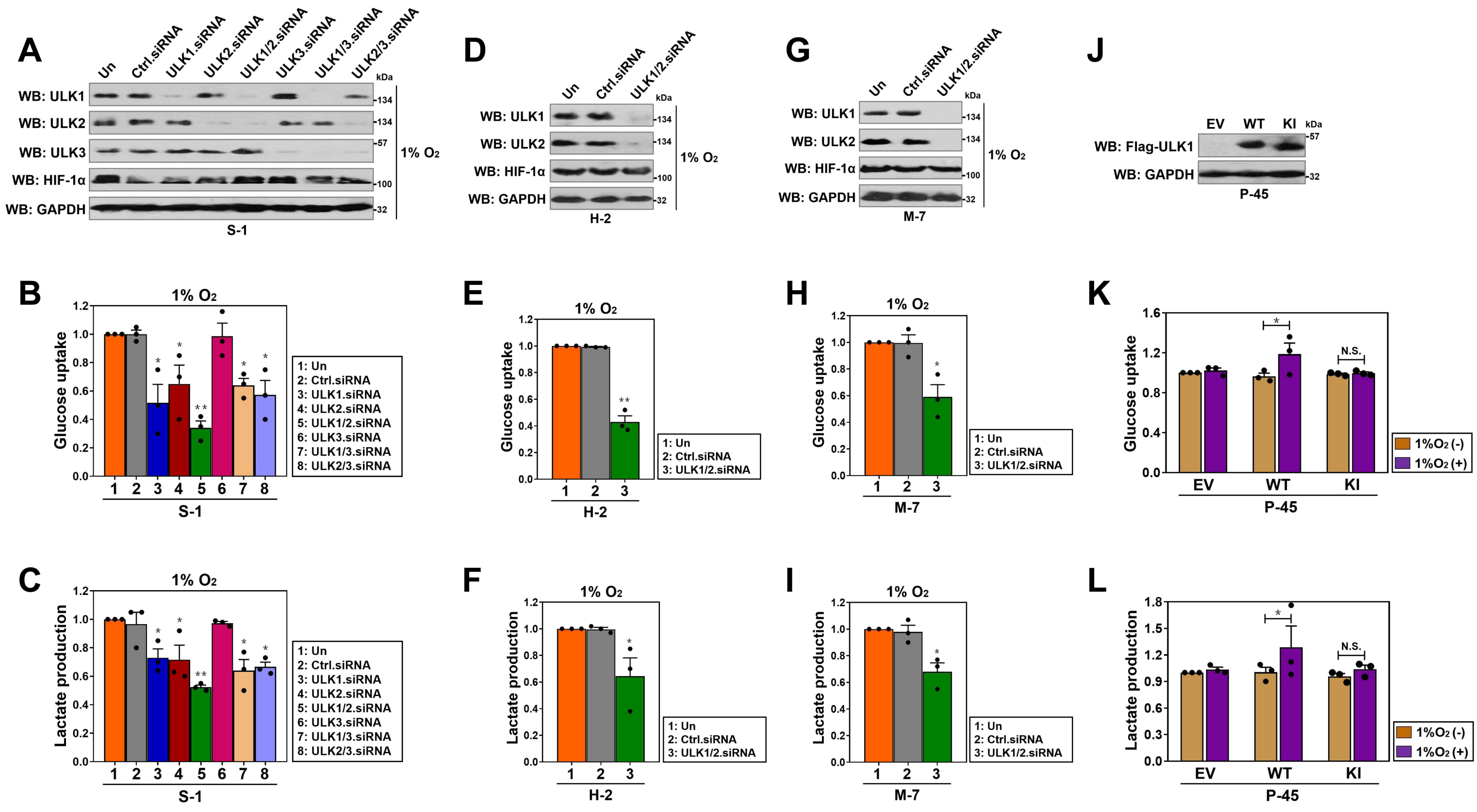
A
Un
Ctrl.siRNA
ULK1.siRNA
ULK2.siRNA
ULK1/2.siRNA
ULK3.siRNA
ULK1/3.siRNA
ULK2/3.siRNA
WB: ULK1
WB: ULK2
WB: ULK3
WB: HIF-1α
WB: GAPDH
1% O2
S-1
D
WB: ULK1
WB: ULK2
WB: HIF-1α
WB: GAPDH
H-2
G
M-7
J
EV
WT
KI
WB: Flag-ULK1
WB: GAPDH
P-45
B
Glucose uptake
1: Un
2: Ctrl.siRNA
3: ULK1.siRNA
4: ULK2.siRNA
5: ULK1/2.siRNA
6: ULK3.siRNA
7: ULK1/3.siRNA
8: ULK2/3.siRNA
C
Lactate production
E
F
H
I
K
L
1%O2 (-)
1%O2 (+)
N.S.

Supplemental Figure 1. ULK1/2, but not ULK3, contributes to the hypoxic glycolysis. (A) Western-blotting examining the levels of ULK1, ULK2 and ULK3 expression in SW-1990 (S-1) cells transfected with siRNAs targeting ULK1, ULK2, ULK3 or both of each other under hypoxia, respectively. Un, Untransfected; Ctrl.siRNA, control siRNA. (B and C) Glucose consumption (B) and lactate production (C) of SW-1990 (S-1) cells transfected with siRNAs targeting ULK1, ULK2, ULK3 or both of each other under hypoxia and of HepG-2 (H-2) and MCF-7 (M-7) cells transfected with siRNAs targeting ULK1 plus ULK2 under hypoxia, respectively. Data are expressed as mean ± s.d. of three independent experiments. *$P < 0.05$ and **$P < 0.01$ versus Un. Two-sided ANOVA with Bonferroni post hoc *t* test correction was used to calculate the *P* value. (D) Western-blotting detecting the levels of ULK1 and ULK2 expression in HepG-2 (H-2) cells transfected with siRNAs targeting ULK1 plus ULK2 under hypoxia, respectively. (E and F) Glucose consumption (E) and lactate production (F) of HepG-2 (H-2) cells transfected with siRNAs targeting ULK1 plus ULK2 under hypoxia. Data are expressed as mean ± s.d. of three independent experiments. *$P < 0.05$ and **$P < 0.01$ versus Un. Two-sided ANOVA with Bonferroni post hoc *t* test correction was used to calculate the *P* value. (G) Western-blotting evaluating the levels of ULK1 and ULK2 expression in MCF-7 (M-7) cells transfected with siRNAs targeting ULK1 plus ULK2 under hypoxia. (H and I) Glucose consumption (H) and lactate production (I) of MCF-7 (M-7) cells transfected with siRNAs targeting ULK1 plus ULK2 under hypoxia. Data are expressed as mean ± s.d. of three independent experiments. *$P < 0.05$ versus Un. Two-sided ANOVA with Bonferroni post hoc *t* test correction was used to calculate the *P* value. (J) Western-blotting determining the levels of Flag-ULK1 expression in PL45 (P-45) cells transfected with Flag-tagged wild-type (WT) or kinase-inactive (KI) mutant ULK1. EV, empty vector. (K and L) Glucose consumption (K) and lactate production (L) of PL45 (P-45) cells transfected with Flag-tagged wild-type (WT) or kinase-inactive (KI) mutant ULK1 in the presence or absence of hypoxia. Data are expressed as mean ± s.d. of three independent experiments. *$P < 0.05$. Two-sided Student's t test was used to calculate the *P* value.

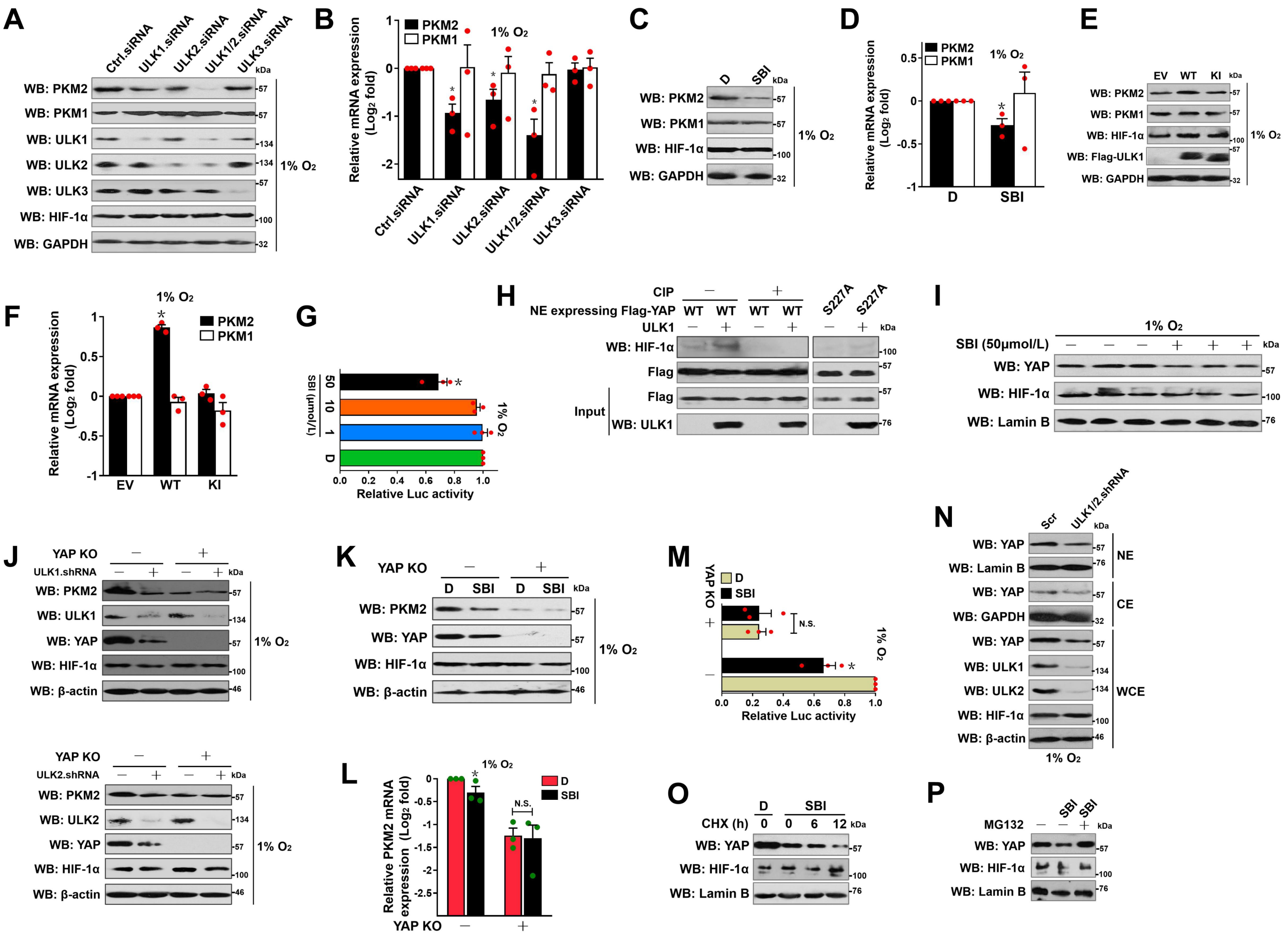
A
B
C
D
E
F
G
H
I
J
K
L
M
N
O
P
1% O2
WB: PKM2
WB: PKM1
WB: ULK1
WB: ULK2
WB: ULK3
WB: HIF-1α
WB: GAPDH
WB: YAP
WB: Lamin B
WB: β-actin
Relative mRNA expression (Log2 fold)
Relative Luc activity
Relative PKM2 mRNA expression (Log2 fold)
YAP KO
SBI
CHX (h)
MG132
NE
CE
WCE

Supplemental Figure 2. Regulatory roles of ULK1/2 in PKM2 transcription and YAP ubiquitylation. (A) Western-blotting examining the abundance of PKM2 and PKM1 expression in SW-1990 cells transfected with ULK1, ULK2, ULK1/2 and ULK3 siRNA under hypoxia, respectively. (B) RT-qPCR analyses of PKM2 and PKM1 gene expression in SW-1990 cells transfected with ULK1, ULK2, ULK1/2 and ULK3 siRNA under hypoxia, respectively. Experiments were performed three times, each with quantitative RT-PCR in technical duplicate and real-time values were normalized to glyceraldehyde 3-phosphate dehydrogenase (GAPDH). Data are expressed as mean ± s.d. *$P < 0.05$ versus Ctrl.siRNA. Two-sided ANOVA with Bonferroni post hoc *t* test correction was used to calculate the *P* value. (C) Western-blotting comparing the levels of PKM2 and PKM1 expression in SW-1990 cells treated with 50 μmol/L SBI-0206965 (SBI) under hypoxia. (D) RT-qPCR analyses of PKM2 and PKM1 gene expression in SW-1990 cells treated with 50 μmol/L SBI-0206965 (SBI) under hypoxia. Experiments were performed three times, each with quantitative RT-PCR in technical duplicate and real-time values were normalized to glyceraldehyde 3-phosphate dehydrogenase (GAPDH). Data are expressed as mean ± s.d. *$P < 0.05$ versus D. Two-sided Student's t test was used to calculate the *P* value. D, DMSO. (E) Western-blotting determining the levels of PKM2 and PKM1 expression in PL45 (P-45) cells transfected with Flag-tagged wild-type (WT) or kinase-inactive (KI) mutant ULK1 under hypoxia. (F) RT-qPCR analyses of PKM2 and PKM1 gene expression in PL45 (P-45) cells transfected with Flag-tagged wild-type (WT) or kinase-inactive (KI) mutant ULK1 under hypoxia. Experiments were performed three times, each with quantitative RT-PCR in technical duplicate and real-time values were normalized to glyceraldehyde 3-phosphate dehydrogenase (GAPDH). Data are expressed as mean ± s.d. *$P < 0.05$ versus EV. Two-sided ANOVA with Bonferroni post hoc *t* test correction was used to calculate the *P* value. EV, empty vector. (G) Luciferase-reporter PKM2 promoter activity analysis of SW-1990 cells treated with or without SBI at the indicated concentrations under hypoxia. Data are expressed as mean ± s.d. of three independent experiments. *$P < 0.05$ versus 0. Two-sided ANOVA with Bonferroni post hoc *t* test correction was

used to calculate the *P* value. (H) *In vitro* protein binding assay with mixing nuclear extraction (NE) from SW-1990 cells expressing Flag-tagged wild-type YAP (WT) or mutant YAP Ser227A (S227A) and purified ULK1 followed by WB analyses of immunoprecipitated Flag with an anti-HIF-1α antibody in the presence or absence of CIP treatment, respectively. (I) Western-blotting detecting the levels of nuclear YAP accumulation in SW-1990 cells with or without SBI (50 μmol/L) under hypoxia. (J) Western-blotting comparing the abundance of PKM2 expression in SW-1990 cells transfected with shRNA targeting ULK1 (top panel) or ULK2 (bottom panel) in the presence or absence of YAP knockout (KO) under hypoxia. (K) Western-blotting assessing the abundance of PKM2 expression in SW-1990 cells with or without SBI (50 μmol/L) treatment in the presence or absence of YAP knockout (KO) under hypoxia. (L) RT-qPCR analyses of PKM2 gene expression in SW-1990 cells SBI (50 μmol/L) treatment in the presence or absence of YAP knockout (KO) under hypoxia, respectively. Experiments were performed three times, each with quantitative RT-PCR in technical duplicate and real-time values were normalized to glyceraldehyde 3-phosphate dehydrogenase (GAPDH). Data are expressed as mean ± s.d. *$P < 0.05$ versus D. Two-sided Student's t test was used to calculate the *P* value. (M) Luciferase-reporter PKM2 promoter activity analysis of SW-1990 cells with or without SBI (50 μmol/L) treatment in the presence or absence of YAP knockout (KO) under hypoxia, respectively. Data are expressed as mean ± s.d. of three independent experiments. *$P < 0.05$ versus D. Two-sided Student's t test was used to calculate the *P* value. (N) Subcellular fractionation analyses examining abundance of nuclear and cytoplasmic YAP protein expression in SW-1990 cells transfected with ULK1/2 shRNA under hypoxia. GAPDH and Lamin B were used as internal control of cytoplasmic and nuclear extractions, respectively. NE, nuclear extraction; CE, cytoplasmic extraction; WCE, whole cell extraction. (O) Western-blotting examining the abundance of nuclear YAP in SW-1990 cells with SBI (50 μmol/L) treatment in the presence or absence of 20 μg/mL CHX for the indicated times. (P) Western-blotting testing the levels of nuclear YAP in SW-1990 cells with SBI (50 μmol/L) treatment in the presence or absence of 10 μmol/L MG132 addition.

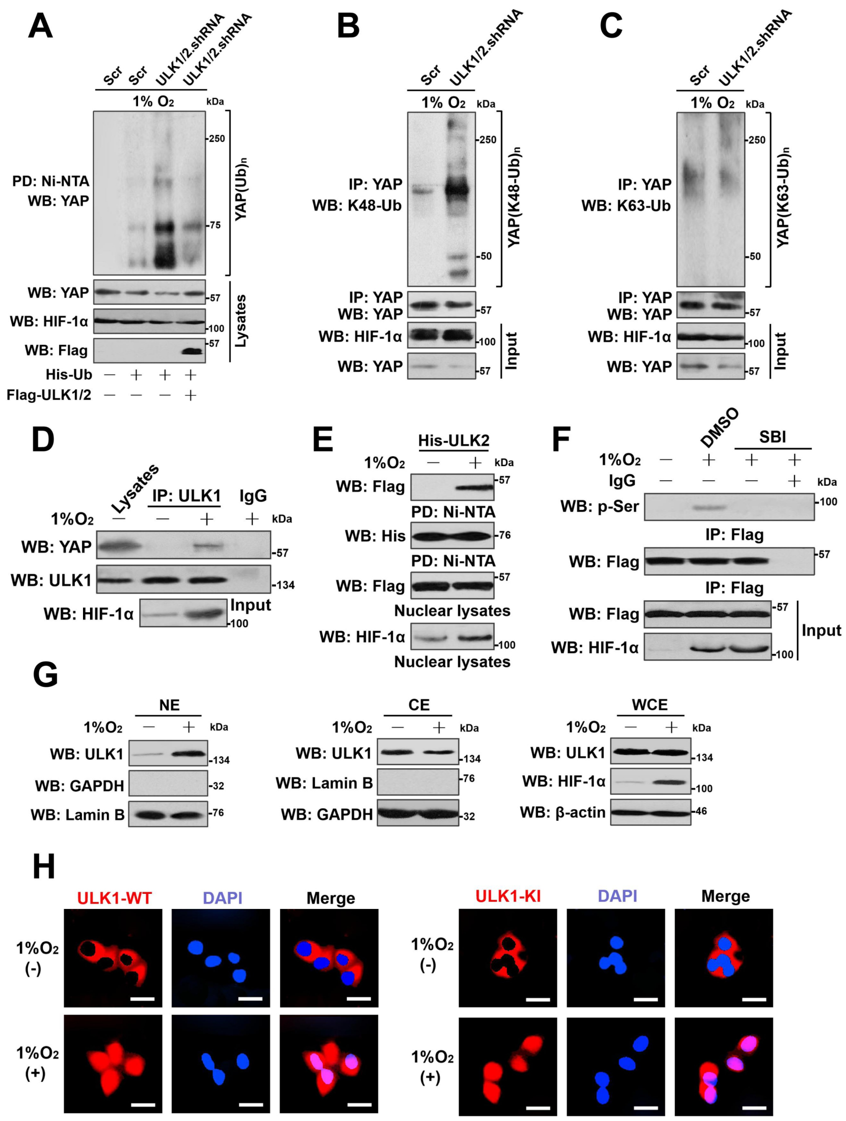
A
Scr
ULK1/2.shRNA
1% O2
kDa
PD: Ni-NTA
WB: YAP
YAP(Ub)n
Lysates
WB: YAP
WB: HIF-1α
WB: Flag
His-Ub
Flag-ULK1/2
B
IP: YAP
WB: K48-Ub
YAP(K48-Ub)n
Input
WB: HIF-1α
C
WB: K63-Ub
YAP(K63-Ub)n
D
Lysates
IP: ULK1
IgG
1%O2
WB: ULK1
E
His-ULK2
WB: His
Nuclear lysates
F
DMSO
SBI
WB: p-Ser
IP: Flag
G
NE
CE
WCE
WB: GAPDH
WB: Lamin B
WB: β-actin
H
ULK1-WT
DAPI
Merge
ULK1-KI
1%O2 (-)
1%O2 (+)

Supplemental Figure 3. Hypoxia evokes ULK1 to interact with and phosphorylate YAP in nucleus. (A) Cellular ubiquitination assays assessing the poly-Ub levels of YAP in SW-1990 cells transfected with ULK1/2 shRNA in the presence of Flag-tagged wild-type ULK1/2 expression under hypoxia. PD, pull-down; Ni-NTA, Ni^{2+}-nitrilotriacetic acid (NTA). (B) Cellular ubiquitination assays detecting the K48-linked Ub levels of YAP in SW-1990 cells transfected with ULK1/2 shRNA under hypoxia. (C) Cellular ubiquitination assays comparing the K63-linked Ub levels of YAP in SW-1990 cells transfected with ULK1/2 shRNA under hypoxia. (D) Coimmunoprecipitation assay comparing the interaction of YAP with ULK1 in SW-1990 cells with or without hypoxia stimuli. (E) His-pulldown assay for determination of ULK2-YAP interaction with mixing purified His-tagged ULK2 immobilized on Ni^{2+}-nitrilotriacetic acid (NTA)-sepharose beads and nuclear lysates from hypoxia-stimulated SW-1990 cells expressing Flag-tagged wild-type YAP followed by WB analyses of proteins on beads with an anti-Flag antibody. PD, pull-down. (F) Coimmunoprecipitation assay examining the abundance of Flag-tagged wild-type YAP phosphorylation in hypoxia-stimulated SW-1990 cells in the presence or absence of 50 μmol/L SBI-0206965 (SBI) treatment with an anti-phospho-serine antibody. (G) Subcellular fractionation analyses assessing ULK1 nuclear localization in SW-1990 cells with or without hypoxia stimuli. (H) Representative immunfluorescence images of Flag-tagged wild-type (WT) or kinase inactive (KI) mutant ULK1 nuclear localization in SW-1990 cells before and after hypoxia stimuli. Scale bar = 20 μm.

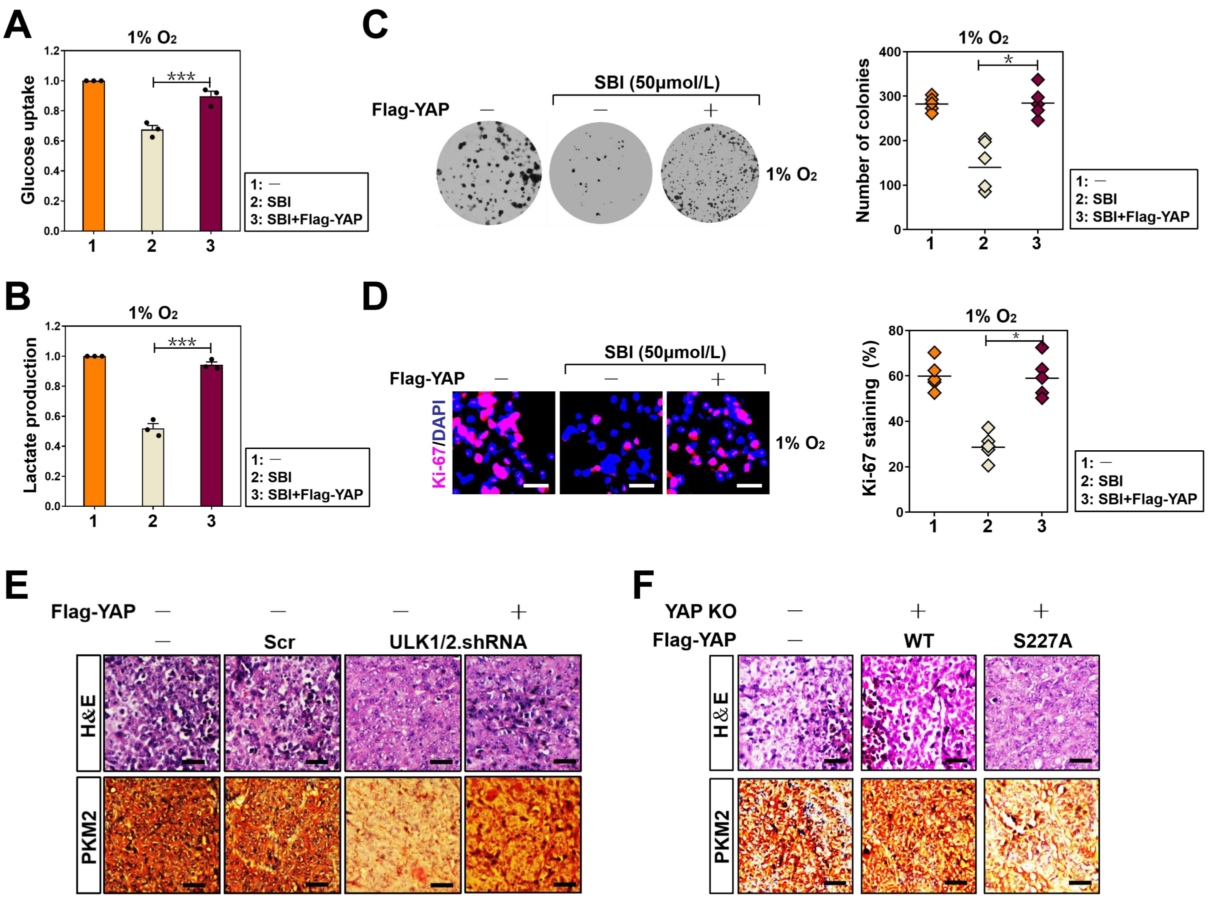
A
1% O2
Glucose uptake
***
1: —
2: SBI
3: SBI+Flag-YAP
C
SBI (50μmol/L)
Flag-YAP
1% O2
Number of colonies
*
B
Lactate production
D
Ki-67/DAPI
Ki-67 staining (%)
E
Flag-YAP
Scr
ULK1/2.shRNA
H&E
PKM2
F
YAP KO
WT
S227A

Supplemental Figure 4. ULK1/2-inducible YAP Ser227 phosphorylation upregulates PKM2 expression of PDAC cells in mice. (A and B) Glucose consumption (A) or lactate production (B) of SBI-0206965 (SBI)-treated SW-1990 cells with or without Flag-tagged wild-type YAP expression under hypoxia. Data are expressed as mean ± s.d. of three independent experiments. ***$P < 0.001$. Two-sided ANOVA with Bonferroni post hoc *t* test correction was used to calculate the *P* value. (C) Colony formation assays of SBI-0206965 (SBI)-treated SW-1990 cells with or without Flag-tagged wild-type YAP expression under hypoxia. Data are expressed as mean ± s.d. of five independent experiments. *$P < 0.05$. Two-sided ANOVA with Bonferroni post hoc *t* test correction was used to calculate the *P* value. (D) Ki-67 staining analyses of SBI-0206965 (SBI)-treated SW-1990 cells with or without Flag-tagged wild-type YAP expression under hypoxia. Data are expressed as mean ± s.d. of five independent experiments. *$P < 0.05$. Two-sided ANOVA with Bonferroni post hoc *t* test correction was used to calculate the *P* value. Scale bar = 100μm. (E) Representative images for H&E and PKM2 staining in mice bearing tumors from the ULK1/2 shRNA-transfected SW-1990 cells with or without Flag-tagged wild-type YAP expression at day 27 after inoculation. Scale bar = 50 μm. (F) Representative images for H&E and PKM2 staining in mice bearing tumors from the YAP knockout (KO) SW-1990 cells with Flag-tagged wild-type YAP (WT) or mutant YAP Ser227A (S227A) reconstitution at day 27 after inoculation. Scale bar = 50 μm.

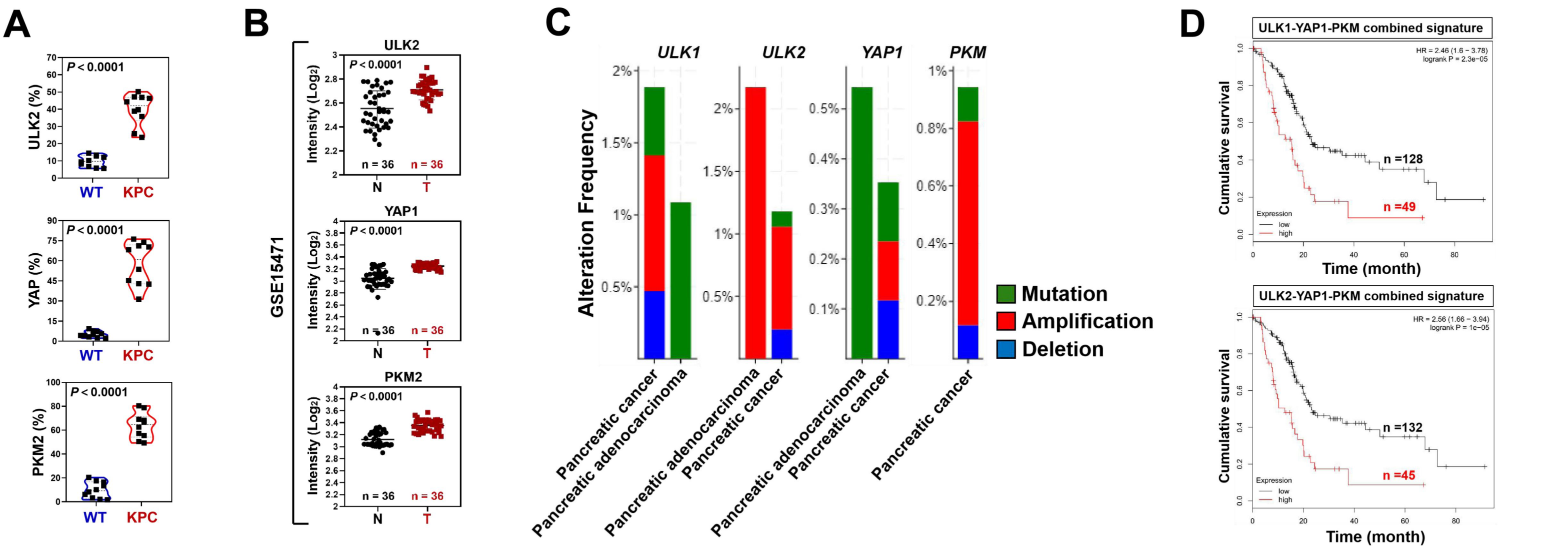

E

		ULK2		Total
		Negative	Positive	
YAP	Negative	25	13	38
	Positive	17	40	57
Total		42	53	95
				P = 0.001

		ULK2		Total
		Negative	Positive	
PKM2	Negative	20	10	30
	Positive	22	43	65
Total		42	53	95
				P = 0.002

		YAP		Total
		Negative	Positive	
PKM2	Negative	20	14	34
	Positive	14	34	48
Total		34	48	82
				P = 0.007

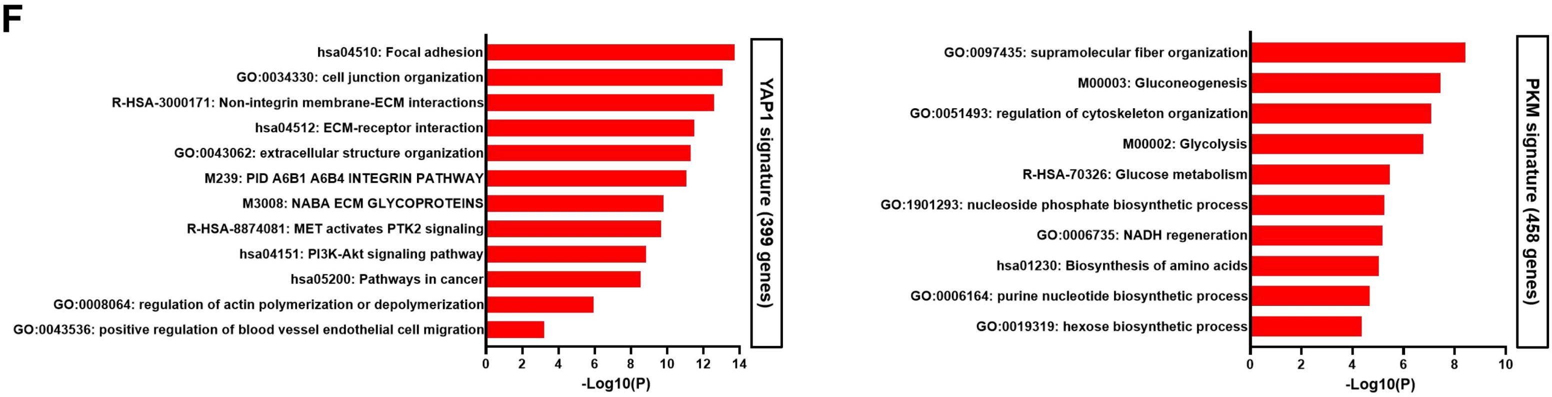

Supplemental Figure 5. Correlation between ULK1/2, YAP and PKM2 in clinical PDAC samples. (A) Quantification of immunohistochemistry comparing expression of ULK2, YAP and PKM2 in pancreas tissues from the wild-type mice and PDAC tissues from the KPC (*Pdx1*-Cre; LSL-$Kras^{G12D/+}$; $Trp53^{fl/+}$) mice as in Figure 6A. (B) Analyses of ULK2, YAP1 and PKM2 mRNA levels in the normal pancreatic tissues and PDAC tumor samples from the publicly available GEO database (GSE15471) containing 36 normal tissues and 36 tumor tissues. (C) Bar graphs showing the frequencies of ULK1, ULK2, YAP1 and PKM genetic alterations in pancreatic tumours. Data are integrated from the cBioPortal (http://www.cbioportal.org/) using the indicated TCGA cancer datasets. (D) Kaplan-Meier curves comparing the overall survival in 177 PDAC patients with low and high expression of the combination of ULK1/2, YAP1 and PKM signature from TCGA datasets. Log-rank test was used to calculate the *P* value. (E) Statistic analyses for ULK2, YAP1 and PKM2 correlation from the IHC staining results in human PDAC tissues. The *P* value shown was calculated by Spearman order correlations. (F) Gene ontology (GO) analyses for YAP1 and PKM signature from CCLE dataset. Annotation clusters are shown according to their enrichment scores.

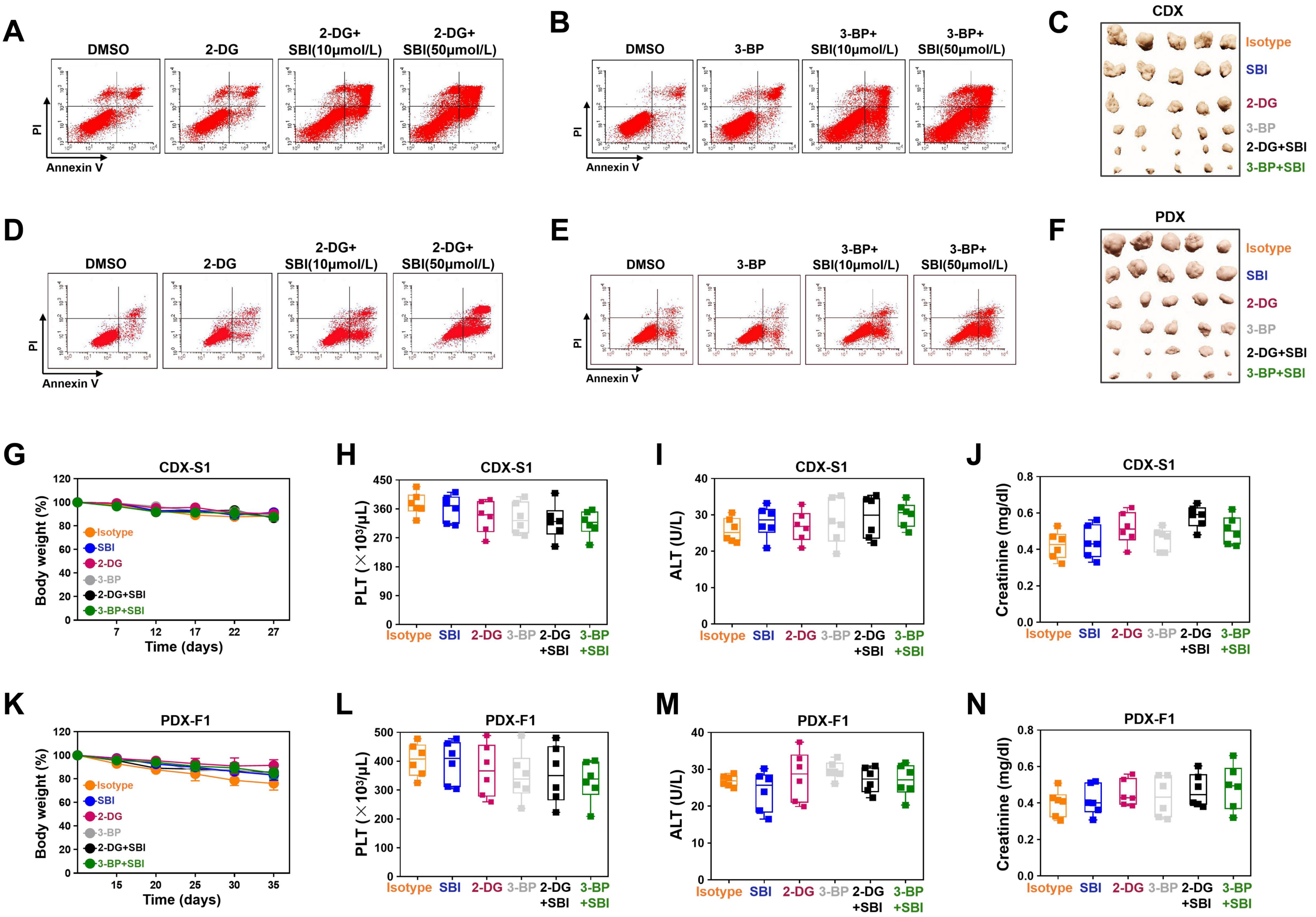
A
DMSO
2-DG
2-DG+ SBI(10μmol/L)
2-DG+ SBI(50μmol/L)
PI
Annexin V
B
DMSO
3-BP
3-BP+ SBI(10μmol/L)
3-BP+ SBI(50μmol/L)
PI
Annexin V
C
CDX
Isotype
SBI
2-DG
3-BP
2-DG+SBI
3-BP+SBI
D
DMSO
2-DG
2-DG+ SBI(10μmol/L)
2-DG+ SBI(50μmol/L)
PI
Annexin V
E
DMSO
3-BP
3-BP+ SBI(10μmol/L)
3-BP+ SBI(50μmol/L)
PI
Annexin V
F
PDX
Isotype
SBI
2-DG
3-BP
2-DG+SBI
3-BP+SBI
G
CDX-S1
Body weight (%)
Time (days)
Isotype
SBI
2-DG
3-BP
2-DG+SBI
3-BP+SBI
H
CDX-S1
PLT (×10³/μL)
I
CDX-S1
ALT (U/L)
J
CDX-S1
Creatinine (mg/dl)
Isotype SBI 2-DG 3-BP 2-DG +SBI 3-BP +SBI
K
PDX-F1
Body weight (%)
Time (days)
Isotype
SBI
2-DG
3-BP
2-DG+SBI
3-BP+SBI
L
PDX-F1
PLT (×10³/μL)
M
PDX-F1
ALT (U/L)
N
PDX-F1
Creatinine (mg/dl)
Isotype SBI 2-DG 3-BP 2-DG +SBI 3-BP +SBI

Supplemental Figure 6. The effects of ULK1/2 deactivation in combination with 2-DG and 3-BP on PDAC cells and mice. (A and B) Representative histograms of flow cytometry with Annexin-V/PI staining in SW-1990 cells treated with 25 mM 2-DG (A) and 100 μM 3-BP (B) with or without SBI-0206965 (SBI) administration. (C) Representative images of xenografts from the tumor-bearing mice as in Figure 7E. (D and E) Representative histograms of flow cytometry with Annexin-V/PI staining in PDC treated with 25 mM 2-DG (D) and 100 μM 3-BP (E) with or without SBI-0206965 (SBI) administration. (F) Representative images of xenografts from the tumor-bearing mice as in Figure 7L. (G-J) Body weight (G), platelets (PLT, H), alanine aminotransferase (ALT, I) and serum creatinine (Scr, J) levels from mice bearing SW-1990 xenografts in the presence of 2-DG or 3-BP in combination with or without SBI-0206965 (SBI) treatment (n=6). Data are presented as mean ± s.d. (K-N) Body weight (K), platelets (PLT, L), alanine aminotransferase (ALT, M) and serum creatinine (Scr, N) levels from mice bearing patient-derived xenografts in the presence of 2-DG or 3-BP in combination with or without SBI-0206965 (SBI) treatment (n=6). Data are presented as mean ± s.d.
